# Chronic Treatment with Serotonin Selective Reuptake Inhibitors Does Not Affect Regrowth of Serotonin Axons Following Amphetamine Injury in the Mouse Forebrain

**DOI:** 10.1523/ENEURO.0444-22.2023

**Published:** 2024-02-09

**Authors:** Haley N. Janowitz, David J. Linden

**Affiliations:** ^1^Cellular and Molecular Medicine Graduate Program, Johns Hopkins University School of Medicine, Baltimore, Maryland 21205; ^2^The Solomon H. Snyder Department of Neuroscience, Johns Hopkins University School of Medicine, Baltimore, Maryland 21205

**Keywords:** brain injury, fluoxetine, regeneration, serotonin, serotonin selective reuptake inhibitors, sertraline

## Abstract

A current hypothesis to explain the limited recovery following brain and spinal cord trauma stems from the dogma that neurons in the mammalian central nervous system lack the ability to regenerate their axons after injury. Serotonin (5-HT) neurons in the adult brain are a notable exception in that they can slowly regrow their axons following chemical or mechanical lesions. This process of regrowth occurs without intervention over several months and results in anatomical recovery that approximates the preinjured state. During development, serotonin is a trophic factor, playing a role in both cell survival and axon growth. Additionally, some studies have shown that stroke patients treated after injury with serotonin selective reuptake inhibitors (SSRIs) appeared to have improved recovery. To test the hypothesis that serotonin can influence the regrowth of 5-HT axons, mice received a high dose of *para*-chloroamphetamine (PCA) to induce widespread retrograde degeneration of 5-HT axons. Then, after a short rest period to avoid any interaction with the acute injury phase, SSRIs were administered daily for 6 or 10 weeks. Using immunohistochemistry in 5-HT transporter-GFP BAC transgenic mice, we determined that while PCA led to a rapid initial decrease in total 5-HT axon length in the somatosensory cortex, visual cortex, or area CA1 of the hippocampus, treatment with either fluoxetine or sertraline (two different SSRIs) did not affect the recovery of axon length. These results suggest that chronic SSRI treatment does not affect the regrowth of 5-HT axons and argue against SSRIs as a potential therapy following brain injury.

## Significance Statement

Brain injuries can result in severe damage to neurons, which are thought to be unable to regrow their axons. Ultimately, this damage can lead to lasting defects in cognition, mood, and sensorimotor function. Limited therapies exist to improve functional outcomes aside from serotonin selective reuptake inhibitors (SSRIs) to reduce associated depression. Some studies suggest that patients treated with SSRIs had functional improvements beyond mood. We investigated the potential of chronic treatment with SSRIs to increase regrowth of serotonin-releasing axons following a selective chemical lesion of those axons in mice. Serotonin axons already have the unusual ability to regrow after injury; however, chronic treatment with SSRIs failed to alter this regrowth, challenging their potential use as therapy after brain injury.

## Introduction

Injuries to the brain and spinal cord often result in permanent sensorimotor and cognitive deficits including paralysis, memory loss, and deficiencies in language. Depression is also often associated with these injuries. A recent study of 559 traumatic brain injury (TBI) patients reported that within 1 year of sustaining a TBI, patients were 7.9 times more likely to develop major depressive disorder (Bombardier et al., 2010). The limited recovery of these behavioral deficits is thought to be a result of damage to axons which are thought to lack the capacity to regrow in the mammalian central nervous system.

Contrary to dogma, serotonin (5-HT) axons regrow in the adult mammalian cortex following amphetamine-induced lesions (O’Hearn et al., 1988; Molliver et al., 1990; Wilson and Molliver, 1994; Mamounas et al., 2000), thermal injury (Hawthorne et al., 2011), or TBIs from stabs (Jin et al., 2016) or cortical impact (Kajstura et al., 2018). Long-term in vivo imaging studies in mice revealed that a stab injury that transected 5-HT axons innervating the neocortex resulted in small regression of the cut axons followed by regrowth of the axons from the cut ends across the glial scar within the stab rift zone (Jin et al., 2016). Furthermore, following *para*-chloroamphetamine (PCA) induced retrograde axonal degeneration of many millimeters, 5-HT axons in the neocortex showed subsequent slow regrowth of axonal density primarily via long-distance regrowth rather than local sprouting (Jin et al., 2016). The regrown 5-HT axons can release serotonin, and regrowth of these axons is associated with improvements in certain behavioral tasks (Jin et al., 2016). The molecular mechanisms of 5-HT axon regrowth have yet to be determined, but this unusual ability may be linked to their long, unmyelinated structure or their use of volume transmission instead of traditional point-to-point synaptic transmission as employed by glutamatergic or GABAergic neurons.

Serotonin is a neuromodulator that regulates mood, appetite, libido, certain aspects of cognition, memory processing, and endocrine systems (Frazer and Hensler, 1999; Kobayashi, 2001). The early embryonic expression of 5-HT receptors and transporters, prior to serotonergic afferents reaching their targets, suggests a role for 5-HT in the developing brain (Lauder, 1990; Vitalis and Parnavelas, 2003; Agrawal et al., 2019). Studies in invertebrates have demonstrated the inhibitory effects of 5-HT on neurite outgrowth and growth cone stability (Haydon et al., 1987; McCobb et al., 1988; van Kesteren and Spencer, 2003), while those performed in mammalian cell culture show a trophic effect of 5-HT (Lauder, 1990; Liu and Lauder, 1991; Lieske et al., 1999; Fricker et al., 2005). Rodent studies showed a necessity for 5-HT or its receptors in the development and organization of thalamocortical axons of the primary somatosensory cortex (van Kleef et al., 2012) and proper innervation of serotonergic axons in the thalamus and hypothalamus (Migliarini et al., 2013).

An experimental therapy for TBI patients is treatment with serotonin selective reuptake inhibitors (SSRIs), which target the serotonin transporter and increase the concentration of serotonin in the CNS (Silver et al., 2009). Meta-analysis of clinical studies has revealed that stroke patients who received poststroke SSRIs as a treatment had better functional recovery than control patients even when the dose of SSRI was subthreshold for relief of depression (Chollet et al., 2011; Mead et al., 2012; Gu and de Wang, 2018; Lin et al., 2018). Another study in patients with mild TBIs showed that treatment with the SSRI sertraline resulted in relief of depression and improved cognitive outcomes (Fann et al., 2001). Yet other studies in both stroke and TBI patients failed to show any functional benefits of treatment with SSRIs (Meythaler et al., 2001; Yue et al., 2017; Legg et al., 2021).

Considering the potential of SSRIs to improve functional recovery in mood, cognition, and motor performance following brain injury and the role of serotonin in axon outgrowth during development, we asked whether chronic treatment with SSRIs positively affects regrowth of serotonin fibers in several regions of the mouse forebrain following injury, measured using immunohistochemistry to identify 5-HT axons. We tested the effect of daily treatments with the SSRI fluoxetine for 6 weeks and 10 weeks at both a low dose and a high dose on total length of 5-HT axons following a chemical lesion. Additionally, we tested the effect of daily treatments with sertraline for 6 weeks. There was no overall effect of SSRIs on total axon length in any of the brain regions or time points we examined, suggesting that chronic treatment with SSRIs does not affect the regrowth of 5-HT axons.

## Materials and Methods

### Animals

All animal procedures were performed in accordance with the Johns Hopkins University animal care and use committee’s regulations. Mice were housed in groups of up to five animals per cage under a 12 h light/dark cycle and were provided with food and water ad libitum. We used both male and female Slc6a4-EGFP BAC transgenic mice created as a part of the GENSAT consortium (line RP23–39F11, BAC BX86, RRID: MMRRC_030692-UCD; Gong et al., 2003). For a second model of serotonin neuron labeling, Slc6a4-Cre BAC transgenic mice created as part of the GENSAT consortium (stock 031028-UCD; RRID: MMRRC_017260-UCD; Gong et al., 2007) were crossed with an mTmG reporter line in which Cre-expressing cells switch from producing membrane-targeted td-Tomato to membrane-targeted GFP fluorophore (Jackson Laboratory, stock #007576, Gt (ROSA)26Sortm4(ACTB-tdTomato,-EGFP)Luo/J, RRID: MGI:3722405; Muzumdar et al., 2007). Both male and female offspring were used for experiments.

### PCA and saline injection protocol

Mice aged 8–15 weeks received PCA (Sigma-Adlrich, catalog #C9635; 20 mg/kg) or saline intraperitoneally. PCA was dissolved in saline at a concentration of 2 mg/ml. Mice received injections twice daily for 4 d. Each day the injections were given 4 h apart (starting at 12 P.M. the first day) with an interval of 16 h between the second injection of 1 d and the first injection of the next. During the 4-day-long injection period, mouse cages were kept at 23–24°C with continuous airflow to minimize PCA-evoked hyperthermia.

### SSRI injection protocol

**Figure 1.**
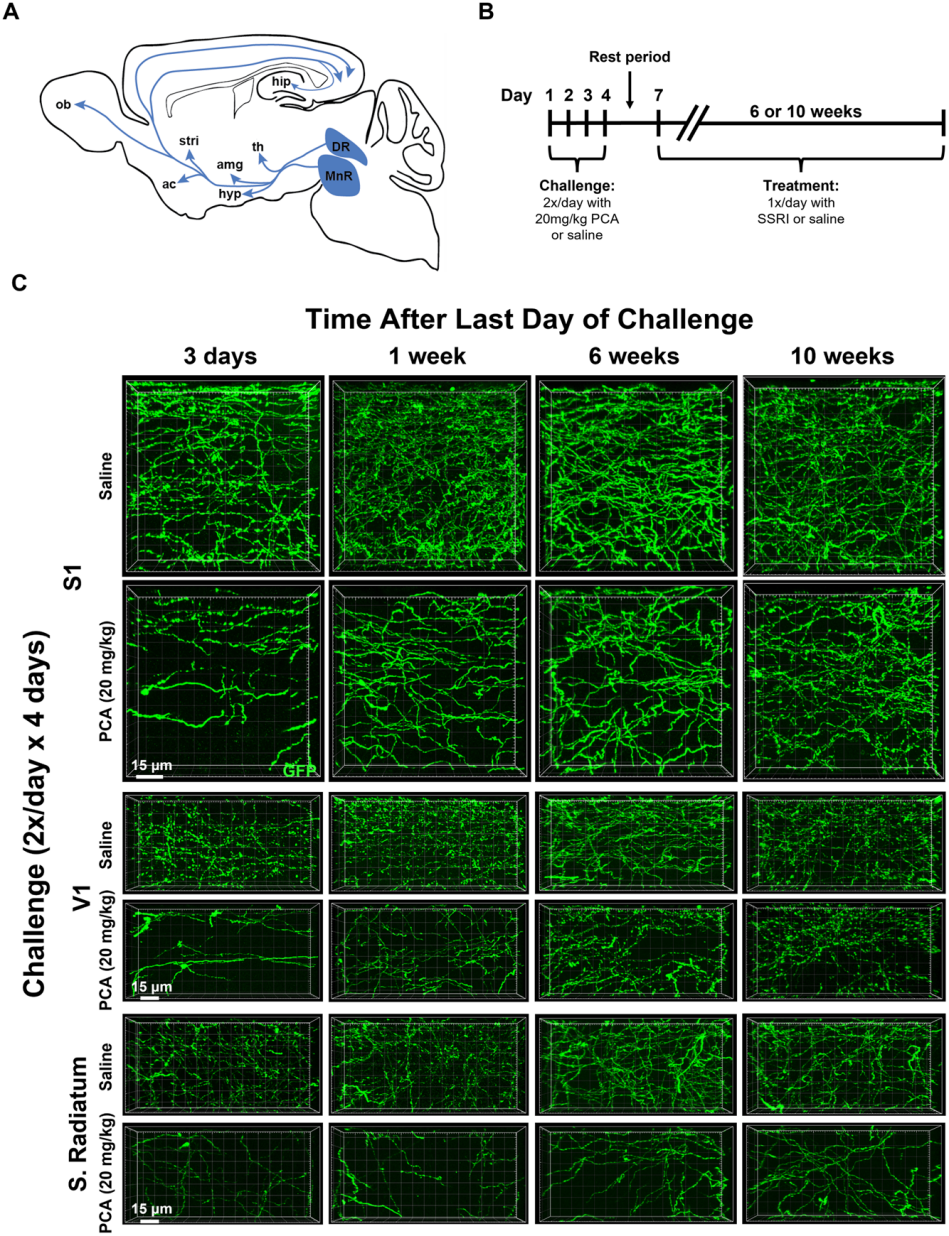
5-HT axons innervating the adult mouse forebrain regrow following a chemical lesion with PCA. ***A***, Schematic diagram of a sagittal section of an adult mouse brain shows some of the diverse projection patterns of the anterior group of 5-HT neurons. Abbreviations: DR, dorsal raphe; MnR, median raphe; th, thalamus; amg,amygdala; hip, hippocampus; hyp, hypothalamus; stri, striatum; ac, nucleus accumbens; and ob, olfactory bulb. ***B***, The experimental timeline shows a PCA/saline challenge period followed, after a 2-day-long rest period, by an SSRI/saline treatment period. ***C***, Representative confocal stack images of anti-GFP fluorescence in layer 1 of primary somatosensory cortex (S1), layer 1 of primary visual cortex (V1), and stratum radiatum of area CA1 of the dorsal hippocampus in sagittal slices from Slc6a4-GFP mice treated with either PCA or saline for 4 d and then allowed to recover for 3 d, 1 week, 6 weeks, or 10 weeks.

Following initial PCA/saline injections and a 2 d recovery period, mice began daily injections of SSRI or saline intraperitoneally (Fig. 1*C*). Fluoxetine (Santa Cruz, catalog #sc-201125A) was dissolved in saline at concentrations of 2 mg/ml and 1 mg/ml and administered to mice at 20 mg/kg or 5 mg/kg, respectively. Sertraline (Acros Organics, catalog #462190010) was dissolved in 1.5% DMSO in saline at a concentration of 1 mg/ml and administered to mice at 10 mg/kg. Saline controls for sertraline animals received 1.5% DMSO in saline. Once daily injections were given for either 6 weeks or 10 weeks depending upon the experiment.

### Immunohistochemistry

Mice were deeply anesthetized with a mixture of ketamine (100 mg/kg) and xylazine (10 mg/kg) and intracardially perfused with ice-cold phosphate-buffered saline (PBS) followed by 4% paraformaldehyde (PFA) in PBS. Brains were removed and postfixed in 4% PFA at 4°C overnight. To cryoprotect the brains, they were incubated in 15% sucrose in PBS for 24 h, followed by 30% sucrose in PBS for an additional 24 h. A freezing sliding microtome (Leica) was used to section whole brains at a 40 µm thickness, and slices were stored in PBS with sodium azide (Fisher, 0.002%) at 4°C until use. Floating brain slices were washed in a buffer of 0.3% Triton X-100 in PBS three times for 5 min each. Slices were then blocked with 5% normal goat serum and 0.3% Triton X-100 in PBS at room temperature for 2 h followed by an incubation with primary antibody in blocking buffer at 4°C overnight. The following primary antibodies were used: mouse anti-NeuN (Millipore ABN60, RRID: AB_2298767; 1:1,000), chicken anti-GFP (Aves Labs GFP-1010, RRID: AB_2307313; 1:6,000), rabbit anti-DCX (Cell Signaling Technology, catalog #4604, RRID: AB_561007; 1:500), and rabbit anti-BDNF (Alomone Labs, catalog #ANT-010, RRID: AB_2039756). The slices were washed in a buffer of 0.3% Triton X-100 in PBS three times for 5 min each and then incubated with secondary antibody in blocking buffer at room temperature for 2 h.

The following secondary antibodies were used: Alexa Fluor 488-labeled goat anti-chicken (Jackson ImmunoResearch Labs, catalog #103-545-155, RRID:AB_2337390; 1:500), Alexa Fluor 594-labeled goat anti-mouse (Jackson ImmunoResearch Labs, catalog #115-586-146, RRID: AB_2338899; 1:500), Alexa Fluor 647-labeled goat anti-mouse (Jackson ImmunoResearch Labs, catalog #115-606-062, RRID:AB_2338925; 1:500), and Alexa Fluor 594-labeled goat anti-rabbit (Jackson ImmunoResearch Labs, catalog #111-585-144, RRID:AB_2307325; 1:500). The slices were washed a final time in a buffer of 0.3% Triton X-100 in PBS three times for 5 min each and then mounted on glass slides which were coverslipped with ProLong Antifade Diamond mounting media. A laser scanning confocal microscope (Zeiss LSM 880; RRID:SCR_020925) was used to acquire *z*-stack images of the primary somatosensory cortex layer 1, primary visual cortex layer 1, stratum radiatum of hippocampal CA1 region or hippocampal CA3 region, and hilus with a 0.5 µm step size using a 40× objective. A different laser scanning confocal microscope (Zeiss LSM 800; RRID: SCR_015963) was used to acquire broader-field tiled, *z*-stack images of area CA1 of the hippocampus with a 1 μm step size using a 20× objective. A wide-field fluorescence microscope (Keyence BZ-X800) was used to acquire *z*-stack images of the hippocampal dentate gyrus with a 1.2 µm step size. Tissue was processed together and imaged using the same settings and laser power by trial. Challenge and treatment groups were blinded during imaging and analysis.

### Image analysis

#### Axon length

Images of the primary somatosensory cortex layer 1 (S1), primary visual cortex layer 1 (V1), and stratum radiatum of the hippocampal CA1 region were acquired using Zeiss Zen Black software (RRID: SCR_018163) as czi files and converted to ims files for analysis in Imaris 9.9 (Bitplane, RRID: SCR_007370). *Z*-stacks were projected as three-dimensional images. S1 was cropped to 512 × 512 × 37 pixels, and V1 and stratum radiatum were cropped to 724 × 362 × 37 pixels. Each cropped image was centered in all dimensions within the original image. The surface function in Imaris was used to create a mask of the visible axons and remove background fluorescence. Total axon length of the mask was then traced using the filament tracing function. A total of three brain slices per animal were analyzed.

#### Doublecortin (DCX) staining

Images of the hippocampal dentate gyrus were acquired using Keyence BZ Analyzer software (RRID: SCR_017205) as tif files and blurring was removed using the Haze Reduction function. Images were then converted to ims files for analysis in Imaris 9.9 (Bitplane, RRID: SCR_007370). The spot function was then used to count the total number of DCX positive cells. Total length of the dentate gyrus was determined using hand-tracing in Fiji (RRID: SCR_002285). A total of three brain slices per animal were analyzed.

#### Brain-derived neurotrophic factor (BDNF) staining

Images of the hippocampal CA3 region and hilus of the dentate gyrus were acquired using Zeiss Zen Black software (RRID: SCR_018163) as czi files. *Z*-stack images were maximally projected and the mean intensity of pixels in the anti-BDNF channel was measured using Fiji (RRID: SCR_002285). A total of three brain slices per animal were analyzed.

### Statistical analysis

Statistical analysis was conducted in R (R Core Team, 2021) with rstatix (Kassambara, 2021) to perform mixed model ANOVA analysis (Kassambara, 2019). All data were tested for normality using the Shapiro–Wilk normality test. Further details of the statistical tests are outlined in Table 1.

**Table 1.** The type of statistical test and the outcome are shown for each group comparison

Figure	Type of test	Statistical analysis
2*B*	Mixed model two-way ANOVA	Interaction: *F*_(1,51)_ = 0.061; *p* = 0.805 PCA effect: *F*_(1,51) _= 76.916; *p* < 0.0001 Fluoxetine effect: *F*_(1,51)_ = 0.311; *p* = 0.58 PCA effect on saline treated: *F*_(1,21)_ = 28.4; *p* < 0.0001 PCA effect on fluoxetine treated: *F*_(1,30)_ = 52.8; *p* < 0.0001 Fluoxetine effect on saline treated: *F*_(1,28)_ = 0.257; *p* = 0.616 Fluoxetine effect on PCA treated: *F*_(1,23)_ = 0.106; *p* = 0.748
2*D*	Mixed model one-way ANOVA	*F*_(1,27)_ = 18.648; *p* < 0.001
3*B*	Mixed model two-way ANOVA	Interaction: *F*_(1,40)_ = 0.371; *p* = 0.546 PCA effect: *F*_(1,40) _= 57.407; *p* < 0.0001 Fluoxetine effect: *F*_(1,40)_ = 1.546; *p* = 0.221 PCA effect on saline treated: *F*_(1,16)_ = 25.741; *p* < 0.001 PCA effect on fluoxetine treated: *F*_(1,24)_ = 37.539; *p* < 0.0001 Fluoxetine effect on saline treated: *F*_(1,19)_ = 1.491; *p* = 0.237 Fluoxetine effect on PCA treated: *F*_(1,21)_ = 0.236; *p* = 0.632
3*D*	Mixed model one-way ANOVA	*F*_(1,18)_ = 7.447; *p* < 0.05
4*B*	Mixed model two-way ANOVA	Interaction: *F*_(1,41)_ = 0.000168; *p* = 0.99 PCA effect: *F*_(1,41) _= 108.954; *p* < 0.0001 Fluoxetine effect: *F*_(1,41)_ = 0.003; *p* = 0.955 PCA effect on saline treated: *F*_(1,19)_ = 73.835; *p* < 0.0001 PCA effect on fluoxetine treated: *F*_(1,22)_ = 46.337; *p* < 0.0001 Fluoxetine effect on saline treated: *F*_(1,22)_ = 0.002; *p* = 0.969 Fluoxetine effect on PCA treated: *F*_(1,19)_ = 0.004; *p* = 0.953
4*D*	Mixed model two-way ANOVA	Interaction: *F*_(1,41)_ = 0.258; *p* = 0.615 PCA effect: *F*_(1,41) _= 35.005; *p* < 0.0001 Fluoxetine effect: *F*_(1,41)_ = 0.241; *p* = 0.626 PCA effect on saline treated: *F*_(1,22)_ = 27.48; *p* < 0.0001 PCA effect on fluoxetine treated: *F*_(1,19)_ = 11.171; *p* < 0.01 Fluoxetine effect on saline treated: *F*_(1,19)_ = 0.574; *p* = 0.458 Fluoxetine effect on PCA treated: *F*_(1,22)_ = 0.000135; *p* = 0.991
5*B*	Mixed model two-way ANOVA	Interaction: *F*_(1,62)_ = 5.531; *p* = 0.022 PCA effect: *F*_(1,62) _= 93.611; *p* < 0.0001 Sertraline effect: *F*_(1,62)_ = 0.214; *p* = 0.645 PCA effect on saline treated: *F*_(1,31)_ = 84.883; *p* < 0.0001 PCA effect on sertraline treated: *F*_(1,31)_ = 23.361; *p* < 0.0001 Sertraline effect on saline treated: *F*_(1,31)_ = 3.151; *p* = 0.086 Sertraline effect on PCA treated: *F*_(1,31)_ = 2.401; *p* = 0.131
5*D*	Mixed model one-way ANOVA	*F*_(1,28)_ = 11.302; *p* < 0.01 (CA3) *F*_(1,28)_ = 7.555, *p* < 0.05 (Hilus)
6*B*	Mixed model two-way ANOVA	Interaction: *F*_(1,51)_ = 0.013; *p* = 0.908 PCA effect: *F*_(1,51) _= 102.741; *p* < 0.0001 Fluoxetine effect: *F*_(1,51)_ = 0.363; *p* = 0.549 PCA effect on saline treated: *F*_(1,22)_ = 36; *p* < 0.0001 PCA effect on fluoxetine treated: *F*_(1,29)_ = 74.3; *p* < 0.0001 Fluoxetine effect on saline treated: *F*_(1,28)_ = 0.218; *p* = 0.644 Fluoxetine effect on PCA treated: *F*_(1,23)_ = 0.202; *p* = 0.657
6*D*	Mixed model two-way ANOVA	Interaction: *F*_(1,39)_ = 0.003; *p* = 0.957 PCA effect: *F*_(1,39) _= 39.019; *p* < 0.0001 Fluoxetine effect: *F*_(1,39)_ = 0.022; *p* = 0.882 PCA effect on saline treated: *F*_(1,15)_ = 12.7; *p* < 0.01 PCA effect on fluoxetine treated: *F*_(1,24)_ = 30.1; *p* < 0.0001 Fluoxetine effect on saline treated: *F*_(1,19)_ = 0.03; *p* = 0.954 Fluoxetine effect on PCA treated: *F*_(1,22)_ = 0.028; *p* = 0.869
7*B*	Mixed model two-way ANOVA	Interaction: *F*_(1,41)_ = 0.074; *p* = 0.787 PCA effect: *F*_(1,41) _= 73.114; *p* < 0.0001 Fluoxetine effect: *F*_(1,41)_ = 0.233; *p* = 0.632 PCA effect on saline treated: *F*_(1,19)_ = 21.8; *p* < 0.001 PCA effect on fluoxetine treated: *F*_(1,22)_ = 70.1; *p* < 0.0001 Fluoxetine effect on saline treated: *F*_(1,22)_ = 0.247; *p* = 0.458 Fluoxetine effect on PCA treated: *F*_(1,19)_ = 0.029; *p* = 0.866
7*D*	Mixed model two-way ANOVA	Interaction: *F*_(1,40)_ = 10.951; *p* = 0.003 PCA effect: *F*_(1,40) _= 38.535; *p* < 0.0001 Fluoxetine effect: *F*_(1,40)_ = 2.975; *p* = 0.092 PCA effect on saline treated: *F*_(1,21)_ = 42.4; *p* < 0.0001 PCA effect on fluoxetine treated: *F*_(1,19)_ = 4.66; *p* = 0.044 Fluoxetine effect on saline treated: *F*_(1,18)_ = 9.64; *p* = 0.006 Fluoxetine effect on PCA treated: *F*_(1,22)_ = 1.66; *p* = 0.211
8*B*	Mixed model two-way ANOVA	Interaction: *F*_(1,58)_ = 1.537; *p* = 0.220 PCA effect: *F*_(1,58) _= 44.891; *p* < 0.0001 Sertraline effect: *F*_(1,58)_ = 5.056; *p* = 0.028 PCA effect on saline treated: *F*_(1,27)_ = 24.4; *p* < 0.0001 PCA effect on sertraline treated: *F*_(1,31)_ = 19.6; *p* < 0.001 Sertraline effect on saline treated: *F*_(1,30)_ = 5.42; *p* = 0.027 Sertraline effect on PCA treated: *F*_(1,28)_ = 0.592; *p* = 0.448
9*C*	Mixed model two-way ANOVA	Interaction: *F*_(1,47)_ = 0.000551; *p* = 0.981 PCA effect: *F*_(1,47) _= 117.649; *p* < 0.0001 Fluoxetine effect: *F*_(1,47)_ = 0.252; *p* = 0.618 PCA effect on saline treated: *F*_(1,21)_ = 75.4; *p* < 0.0001 PCA effect on fluoxetine treated: *F*_(1,26)_ = 54.5; *p* < 0.0001 Fluoxetine effect on saline treated: *F*_(1,25)_ = 0.111; *p* = 0.742 Fluoxetine effect on PCA treated: *F*_(1,22)_ = 0.152; *p* = 0.700
9*E*	Mixed model two-way ANOVA	Interaction: *F*_(1,37)_ = 1.575; *p* = 0.217 PCA effect: *F*_(1,37) _= 71.606; *p* < 0.0001 Fluoxetine effect: *F*_(1,37)_ = 1.722; *p* = 0.197 PCA effect on saline treated: *F*_(1,15)_ = 28.5; *p* < 0.0001 PCA effect on fluoxetine treated: *F*_(1,22)_ = 51.2; *p* < 0.0001 Fluoxetine effect on saline treated: *F*_(1,17)_ = 2.39; *p* = 0.141 Fluoxetine effect on PCA treated: *F*_(1,20)_ = 0.002; *p* = 0.962
10*B*	Mixed model two-way ANOVA	Interaction: *F*_(1,40)_ = 0.509; *p* = 0.480 PCA effect: *F*_(1,40) _= 105.082; *p* < 0.0001 Fluoxetine effect: *F*_(1,40)_ = 0.019; *p* = 0.891 PCA effect on saline treated: *F*_(1,18)_ = 257; *p* < 0.0001 PCA effect on fluoxetine treated: *F*_(1,22)_ = 138; *p* < 0.0001 Fluoxetine effect on saline treated: *F*_(1,21)_ = 0.13; *p* = 0.722 Fluoxetine effect on PCA treated: *F*_(1,19)_ = 0.56; *p* = 0.463
10*D*	Mixed model two-way ANOVA	Interaction: *F*_(1,41)_ = 0.172; *p* = 0.680 PCA effect: *F*_(1,41) _= 105.082; *p* < 0.0001 Fluoxetine effect: *F*_(1,41)_ = 0.000916; *p* = 0.976 PCA effect on saline treated: *F*_(1,22)_ = 65.9; *p* < 0.0001 PCA effect on fluoxetine treated: *F*_(1,19)_ = 41.8; *p* < 0.0001 Fluoxetine effect on saline treated: *F*_(1,19)_ = 0.083; *p* = 0.776 Fluoxetine effect on PCA treated: *F*_(1,22)_ = 0.088; *p* = 0.769
10*F*	Mixed model two-way ANOVA	Interaction: *F*_(1,61)_ = 3.028; *p* = 0.087 PCA effect: *F*_(1,61) _= 120.55; *p* < 0.0001 Sertraline effect: *F*_(1,61)_ = 0.034; *p* = 0.855 PCA effect on saline treated: *F*_(1,30)_ = 73.5; *p* < 0.0001 PCA effect on sertraline treated: *F*_(1,31)_ = 47.2; *p* < 0.0001 Sertraline effect on saline treated: *F*_(1,31)_ = 1.28; *p* = 0.266 Sertraline effect on PCA treated: *F*_(1,30)_ = 1.74; *p* = 0.196

## Results

### Chronic fluoxetine treatment does not affect 5-HT axon regrowth in adult mouse primary somatosensory cortex

Serotonergic (5-HT) axons that innervate the forebrain arise from somata located in the brainstem raphe nuclei, including the dorsal raphe (DR) and median raphe (MnR), and project anteriorly before turning dorsally and finally posteriorly to innervate the cortex, creating a C-shape when viewed in the sagittal plane (Fig. 1A; Tork, 1990; Vitalis et al., 2013). Using Slc6a4-GFP BAC transgenic mice that express GFP under the 5-HT transporter (SERT), we have visualized 5-HT axons using anti-GFP immunocytochemistry. Previous work has shown that these GFP-immunoreactive somata and axons are indeed serotonergic as indicated by coimmunostaining for SERT, tryptophan hydroxylase 2 (TPH2), and 5-HT itself (Jin et al., 2016; Kajstura et al., 2018). Three days after treatment with PCA, the total length of GFP-positive 5-HT axons in layer 1 of the primary somatosensory cortex, layer 1 of the primary visual cortex, and the stratum radiatum of area CA1 of the dorsal hippocampus were substantially reduced compared with saline controls, and this measure slowly returned over the course of weeks (Fig. 1*C*) as previously described in both mice (Jin et al., 2016) and rats (O’Hearn et al., 1988; Molliver et al., 1990; Wilson and Molliver, 1994; Mamounas et al., 2000).

To test the hypothesis that chronic treatment with SSRIs affects 5-HT axon regrowth, we challenged Slc6a4-GFP mice with PCA twice daily for 4 d, and then following a 2 d rest period, we treated them once daily with intraperitoneal injections of an SSRI or saline control for either 6 or 10 weeks (Fig. 1*B*). The rest period was important because we sought to test the effect of SSRIs on the recovery process and sought to diminish interactions between SSRI treatment and the acute injury phase evoked by PCA. We selected the 6 or 10 week duration of the treatment period to match a time point during recovery where some axon regrowth has occurred, but the density of axons has yet to reach its maximal extent (Jin et al., 2016).

Slc6a4-GFP mice between the ages of 2 and 3.5 months were assigned to receive either 20 mg/kg of PCA or an equal volume of saline for their challenge period. Mice were first separated by age and sex, and then each group was randomly assigned to a challenge group to ensure similar distribution of both sex and age among challenge groups (PCA vs saline). Mice within each group were then further assigned to receive either 20 mg/kg of the SSRI fluoxetine or an equal volume of saline for their treatment period, again separating by age and sex prior to random assignment to treatment group to ensure similar distribution of both sex and age among treatment groups. This experiment was not designed to have the statistical power to resolve sex as a biological variable.

Both PCA and fluoxetine were dissolved in saline, and all injections were delivered intraperitoneally. At the conclusion of each trial, mice were killed, and their brains were fixed and sliced in the sagittal plane. Slices were then immuno-labeled with antibodies against GFP to amplify the endogenous GFP expressed in 5-HT axons and with antibodies against NeuN to identify neuronal nuclei, in order to distinguish cortical layers. Three slices per animal were selected from similar regions within either brain hemisphere (between 1.5–2 mm from the midline) that included the primary somatosensory cortex (S1). Images of layer 1 of S1 were acquired, and total axon length was measured using exhaustive tracing of GFP-labeled fibers in 3D projected confocal stacks cropped to an equivalent 3D volume.

**Figure 2.**
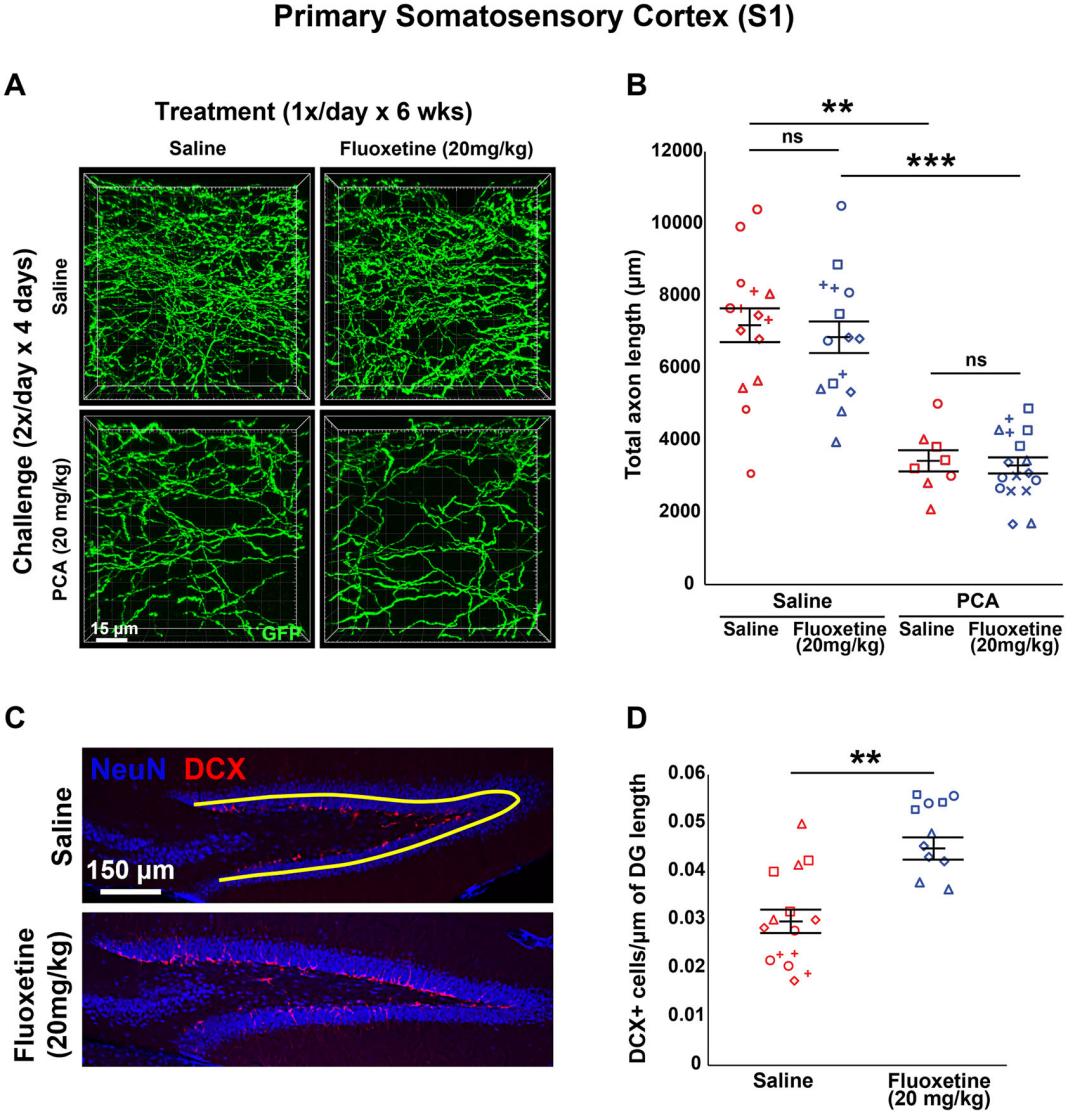
Daily injections of 20 mg/kg fluoxetine for 6 weeks following injury with PCA does not affect 5-HT axon regrowth in layer 1 of somatosensory cortex (S1). *A*, *B*, Slc6a4-GFP mice were initially treated with either PCA or saline for 4 d, allowed 2 d of recovery, and then followed by daily injections of either fluoxetine or saline for 6 weeks. ***A***, Representative images of each combination of conditions. ***B***, Total axon length was determined via histological analysis. Images were acquired using confocal microscopy and layer 1 of area S1 was analyzed in 3D with IMARIS software. Each animal had three technical replicates to account for within-animal variability. Each animal is represented by a different plot symbol. Mean and SE are plotted. Group data were analyzed using a mixed effect repeated-measures ANOVA. *N* = 9 males and 10 females. *F*_(1,30)_ = 52.8, *p* < 0.0001 for the effect of PCA in fluoxetine treated animals. *F*_(1,21)_ = 28.4, *p* < 0.0001 for the effect of PCA in saline-treated animals. *F*_(1,23)_ = 0.106, *p* = 0.748 for the effect of fluoxetine in PCA treated animals. *F*_(1,28)_ = 0.257, *p* = 0.616 for the effect of fluoxetine in saline-treated animals. ***C***, ***D***, Total number of DCX immuno-positive cells in the DG were measured using histological analysis. ***C***, Representative images of each condition with an example tracing of DG length in yellow. ***D***, Images were acquired using widefield microscopy and analyzed with IMARIS. The total number of DCX immune-positive cells was normalized to the length of the DG to account for small variations in the medial–lateral location of each sagittal slice. Each animal had three technical replicates to account for within-animal variability. Each animal is represented by a different plot symbol. Mean and SE are plotted. Group data were analyzed using a mixed effect repeated-measures ANOVA. *F*_(1,27)_ = 18.648, *p* < 0.001.

After 6 weeks of treatment, there was a main effect of PCA (*F*_(1,51) _= 76.916; *p* < 0.0001) where mice that received PCA during their challenge period had a significantly smaller total axon length compared with saline controls regardless of treatment (*F*_(1,21)_ = 28.4, *p* < 0.0001 for saline treated; *F*_(1,30)_ = 52.8, *p* < 0.0001 for fluoxetine treated; Fig. 2*A*,*B*). Importantly, there was not a main effect of 20 mg/kg of fluoxetine treatment for 6 weeks (*F*_(1,51)_ = 0.311; *p* = 0.58; Fig. 2*A*,*B*). Fluoxetine did not affect total axon length in animals initially challenged with PCA (*F*_(1,23)_ = 0.106; *p* = 0.748; Fig. 2*A*,*B*) or saline (*F*_(1,28)_ = 0.257; *p* = 0.616; Fig. 2*A*,*B*) compared with saline controls.

Considering the action of SSRIs on the 5-HT transporter, it is feasible that treating Slc6a4-GFP mice with SSRIs could affect expression of 5-HT transporter promoter-driven expression of GFP over time. For this reason, we repeated this experiment in a different mouse line, Slc6a4-Cre:mTmG, which expresses membrane-bound GFP only in 5-HT transporter-driven Cre-positive cells. In these mice, the Cre-mediated excision event that evokes GFP expression is induced prior to exposure to SSRIs and leaves GFP expression under the control of a strong viral promoter, not the promoter of the 5-HT transporter. After 6 weeks of treatment with 20 mg/kg fluoxetine once daily, there was a main effect of PCA (*F*_(1,55) _= 47.882, *p* < 0.0001; *F*_(1,27)_ = 5.135, *p* < 0.05 for saline treated; *F*_(1,28)_ = 78.268, *p* < 0.0001 for fluoxetine treated), but no significant main effect of fluoxetine on total axon length (*F*_(1,55) _= 1.751, *p* = 0.191; *F*_(1,30)_ = 10.885, *p* = 0.003 for saline treated; *F*_(1,25)_ = 1.634, *p* = 0.213 for PCA treated). This indicates that the effects of PCA challenge and fluoxetine treatment that we observed in Figure 2 are unlikely to be confounded by GFP expression effects in 5-HT axons.

Chronic fluoxetine administration has been shown to increase DCX immunoreactivity in the dentate gyrus of the hippocampus in rodents (Navailles et al., 2008; Wang et al., 2011; Klomp et al., 2014; Sachs and Caron, 2014). To ensure our method of fluoxetine delivery was effective, we measured the number of DCX-positive cells in the hippocampal dentate gyrus. Animals treated with fluoxetine for 6 weeks had significantly more DCX-positive cells normalized to the total length of the dentate gyrus compared with those treated with saline (*F*_(1,27)_ = 18.648; *p* < 0.001; Fig. 2*C*,*D*).

**Figure 3.**
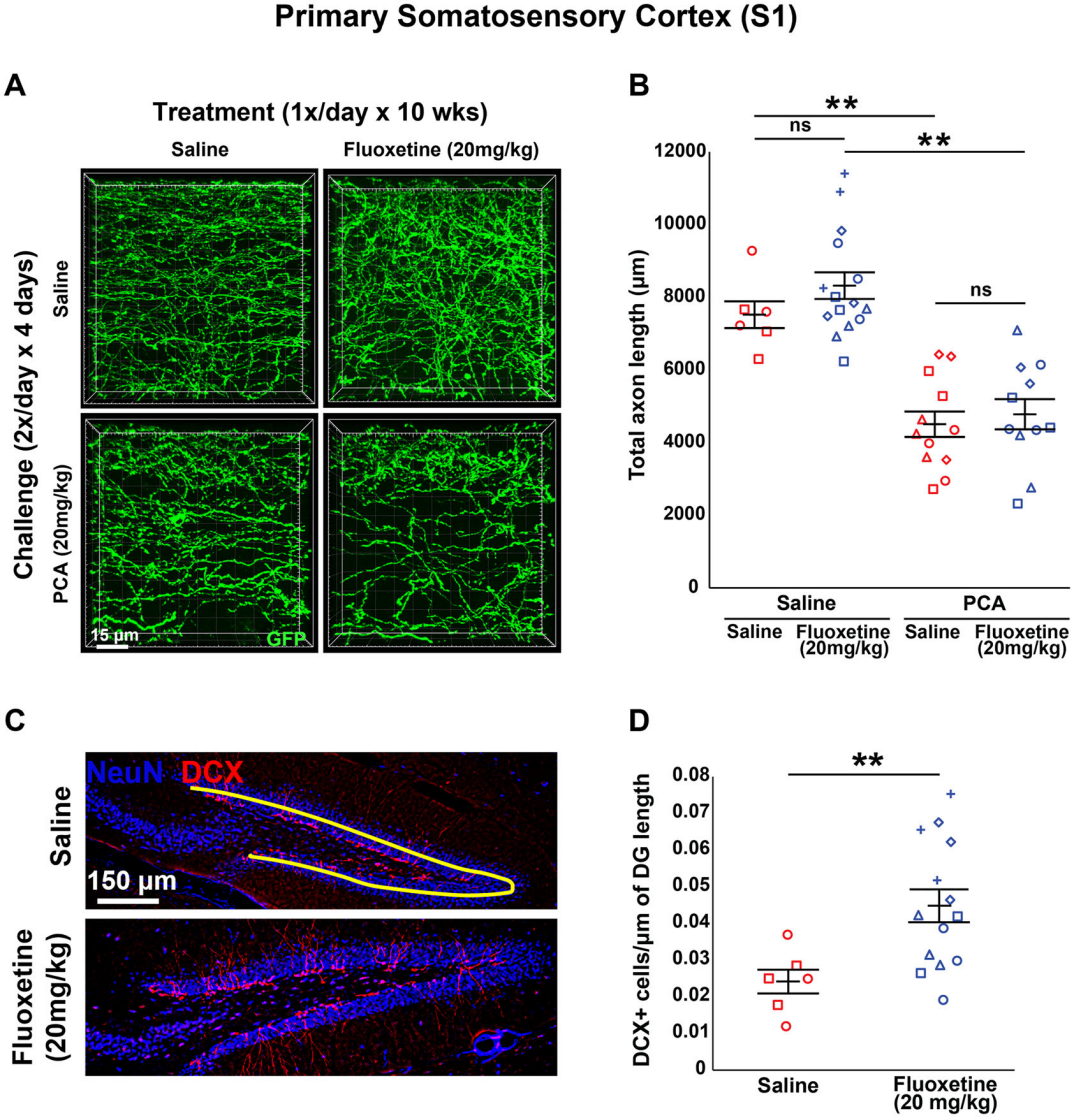
Daily injections of 20 mg/kg fluoxetine for 10 weeks following injury with PCA does not affect 5-HT axon regrowth in layer 1 of primary somatosensory cortex. ***A***, ***B***, Slc6a4-GFP mice were initially treated with either PCA or saline for 4 d, allowed 2 d of recovery, and then followed by daily injections of either fluoxetine or saline for 10 weeks. ***A***, Representative images of each combination of conditions. ***B***, Each animal is represented by a different plot symbol. Mean and SE are plotted. Group data were analyzed using a mixed effect repeated-measures ANOVA. *N* = 3 males and 12 females. *F*_(1,24)_ = 37.5, *p* < 0.0001 for the effect of PCA in fluoxetine treated animals. *F*_(1,16)_ = 25.7, *p* < 0.001 for the effect of PCA in saline-treated animals. *F*_(1,21)_ = 0.236, *p* = 0.632 for the effect of fluoxetine in PCA-treated animals. *F*_(1,19)_ = 1.419, *p* = 0.237 for the effect of fluoxetine in saline-treated animals. ***C***, ***D***, Total number of DCX immuno-positive cells in the DG were measured using histological analysis. ***C***, Representative images of each condition with an example tracing of DG length in yellow. ***D***, Each animal is represented by a different plot symbol. Mean and SE are plotted. Group data were analyzed using a mixed effect repeated-measures ANOVA. *F*(1,18) = 7.447, *p* < 0.05.

To determine whether longer treatment was necessary to see an effect of fluoxetine treatment, we applied 20 mg/kg fluoxetine for 10 weeks. Similar to the 6 week treatment, there was a main effect of PCA (*F*_(1,40) _= 57.407; *p* < 0.0001) where mice that received PCA during their challenge period had a significantly smaller total axon length compared with saline controls regardless of treatment (*F*_(1,16)_ = 25.741, *p* < 0.001 for saline treated; *F*_(1,24)_ = 37.539, *p* < 0.0001 for fluoxetine treated; Fig. 3*A*,*B*). Additionally, there was still no main effect of treatment with 20 mg/kg fluoxetine (*F*_(1,40)_ = 1.546; *p* = 0.221; Fig. 3*A*,*B*) with no increase in total axon length in animals initially challenged with PCA (*F*_(1,21)_ = 0.236; *p* = 0.632; Fig. 3*A*,*B*) or saline (*F*_(1,19)_ = 1.419; *p* = 0.237; Fig. 3*A*,*B*) compared with saline controls. Once again, we validated our method of fluoxetine delivery for the extended treatment period of 10 weeks and found that animals treated with fluoxetine had significantly more DCX-positive cells normalized to the total length of the dentate gyrus compared with those treated with saline (*F*_(1,18)_ = 7.447; *p* < 0.05; Fig. 3*C*,*D*).

**Figure 4.**
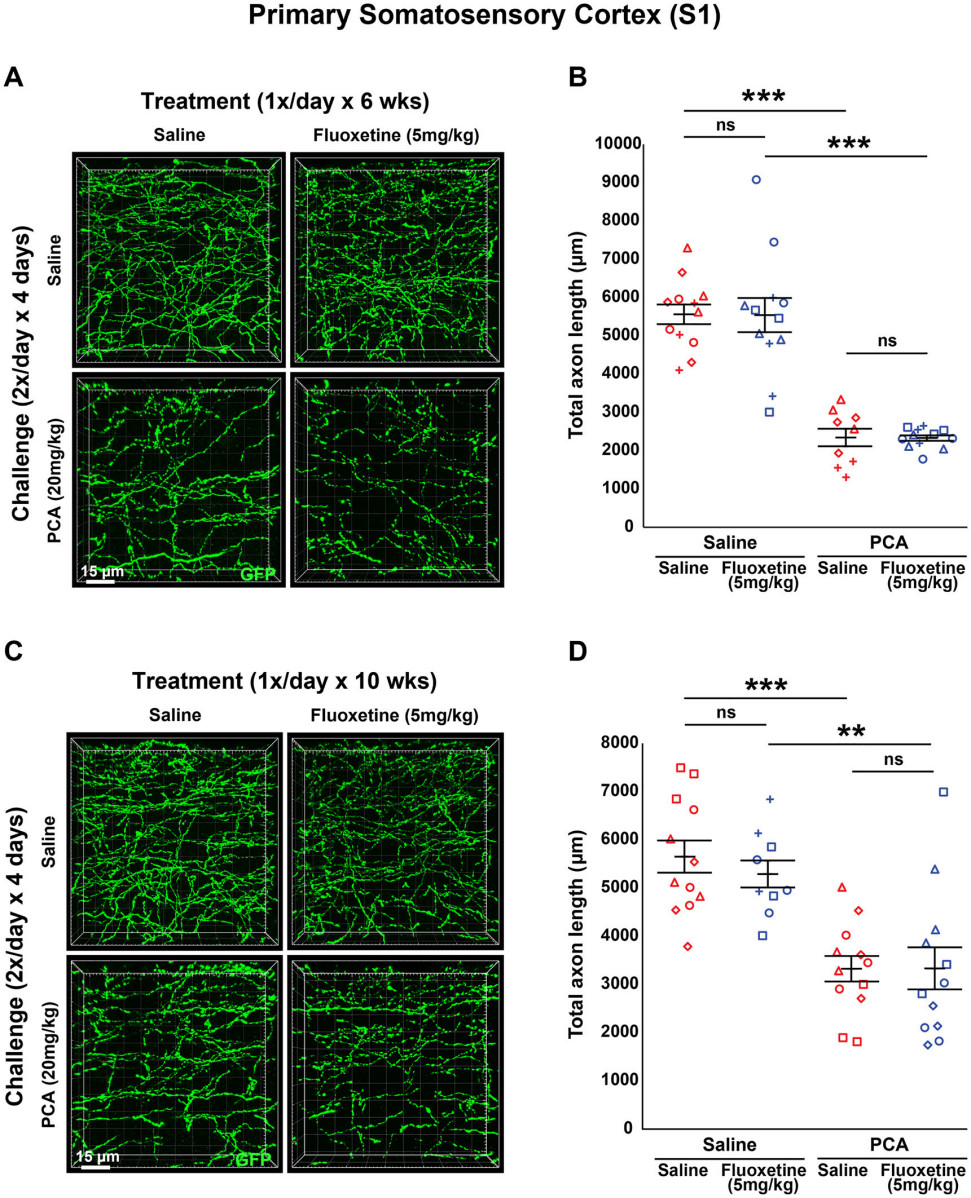
Daily injections of 5 mg/kg fluoxetine following injury with PCA do not affect 5-HT axon regrowth in layer 1 of primary somatosensory cortex. ***A***, ***B***, Slc6a4-GFP mice were initially treated with either PCA or saline for 4 d, allowed 2 d of recovery, and then followed by daily injections of either fluoxetine or saline for 6 weeks. ***A***, Representative images of each combination of conditions. ***B***, Each animal is represented by a different plot symbol. Mean and SE are plotted. Group data were analyzed using a mixed effect repeated-measures ANOVA. *N* = 5 males and 10 females. *F*_(1,22)_ = 37.539, *p* < 0.0001 for the effect of PCA in fluoxetine-treated animals. *F*_(1,19)_ = 73.835, *p* < 0.0001 for the effect of PCA in saline-treated animals. *F*_(1,19)_ = 0.004, *p* = 0.953 for the effect of fluoxetine in PCA-treated animals. *F*_(1,22)_ = 0.002, *p* = 0.969 for the effect of fluoxetine in saline-treated animals. ***C***, ***D***, Slc6a4-GFP mice were initially treated with either PCA or saline for 4 d, allowed 2 d of recovery, and then followed by daily injections of either fluoxetine or saline for 10 weeks. ***C***, Representative images of each combination of conditions. ***D***, Each animal is represented by a different plot symbol. Mean and SE are plotted. Group data were analyzed using a mixed effect repeated-measures ANOVA. *N* = 6 males and 9 females. *F*_(1,19)_ = 11.171, *p* < 0.01 for the effect of PCA in fluoxetine treated animals. *F*_(1,22)_ = 27.48, *p* < 0.0001 for the effect of PCA in saline-treated animals. *F*_(1,22)_ = 0.000135, *p* = 0.991 for the effect of fluoxetine in PCA-treated animals. *F*_(1,19)_ = 0.574, *p* = 0.458 for the effect of fluoxetine in saline-treated animals.

A once daily dosage of 20 mg/kg of fluoxetine is at the top of the typical range of dosage when studying its effects on neurogenesis and anxiety/mood responses in rodents (Hodes et al., 2010, 2009; Karpova et al., 2009; Marlatt et al., 2010). In some studies which examined neuroprotection and neurogenesis in rodents, a lower dose was more effective than a higher dose (Zhu et al., 2012; Pawluski et al., 2014). With this in mind, we assessed a lower dose of 5 mg/kg fluoxetine, once daily, for both a 6 week and a 10 week treatment to determine its effectiveness in promoting axon regrowth. We still observed a main effect of PCA after 6 weeks of treatment (*F*_(1,41) _= 108.954, *p* < 0.0001; *F*_(1,19)_ = 73.835, *p* < 0.0001 for saline treated; *F*_(1,22)_ = 37.539, *p* < 0.0001 for fluoxetine treated; Fig. 4*A*,*B*) and 10 weeks of treatment (*F*_(1,41) _= 35.005, *p* < 0.0001; *F*_(1,22)_ = 27.48, *p* < 0.0001 for saline treated; *F*_(1,19)_ = 11.171, *p* < 0.01 for fluoxetine treated; Fig. 4*C*,*D*). Similar to the higher dose of fluoxetine, there was no main effect of treatment with 5 mg/kg fluoxetine after 6 weeks of treatment (*F*_(1,41) _= 0.003, *p* = 0.955; *F*_(1,22)_ = 0.002, *p* = 0.969 for saline treated; *F*_(1,19)_ = 0.004, *p* = 0.953 for PCA treated; Fig. 4*A*,*B*) or 10 weeks of treatment (*F*_(1,41) _= 0.241, *p* = 0.626; *F*_(1,19)_ = 0.574, *p* = 0.458 for saline treated; *F*_(1,22)_ = 0.000135, *p* = 0.991 for PCA treated; Fig. 4*C*,*D*). Since neither length of treatment nor dosage of fluoxetine changed total 5-HT axon length after a chemical lesion with PCA, we can conclude that in our model of injury chronic treatment with the SSRI fluoxetine does not have a positive effect on the regrowth of 5-HT axons in the primary somatosensory cortex.

### Chronic sertraline treatment does not affect 5-HT axon regrowth in adult mouse primary somatosensory cortex

**Figure 5.**
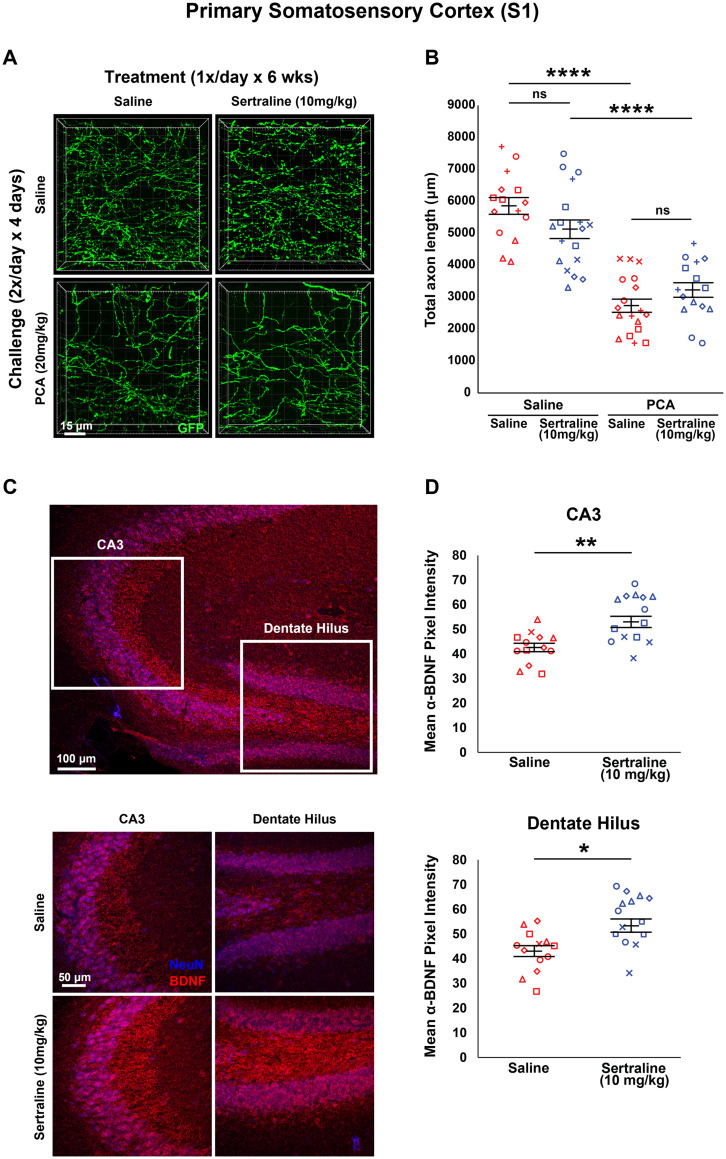
Daily injections of 10 mg/kg sertraline following injury with PCA do not affect 5-HT axon regrowth in layer 1 of primary somatosensory cortex. ***A***, ***B***, Slc6a4-GFP mice were initially treated with either PCA or saline for 4 d, allowed 2 d of recovery, and then followed by daily injections of either sertraline or saline for 6 weeks. ***A***, Representative images of each combination of conditions. ***B***, Each animal is represented by a different plot symbol. Mean and SE are plotted. Group data were analyzed using a mixed effect repeated-measures ANOVA. *N* = 8 males and 14 females. *F*_(1,31)_ = 23.361, *p* < 0.0001 for the effect of PCA in fluoxetine-treated animals. *F*_(1,31)_ = 84.883, *p* < 0.0001 for the effect of PCA in saline-treated animals. *F*_(1,31)_ = 2.401, *p* = 0.131 for the effect of fluoxetine in PCA-treated animals. *F*_(1,31)_ = 3.151, *p* = 0.086 for the effect of fluoxetine in saline-treated animals. ***C***, ***D***, Intensity of BDNF immunoreactivity in the CA3 region of the hippocampus and the hilus of the dentate gyrus was measured using histological analysis. ***C***, Representative images of each condition in each brain region. Location of each region outlined in a lower magnification image. ***D***, Images were acquired using confocal microscopy and the mean pixel intensity of the anti-BDNF channel was measured using ImageJ software. Each animal had three technical replicates to account for within-animal variability. Each animal is represented by a different plot symbol. Mean and SE are plotted. Group data were analyzed using a mixed effect repeated-measures ANOVA. *F*_(_1,18) = 7.447, *p* < 0.05. *F*_(1,28)_ = 11.302, *p* < 0.01 for the effect on BDNF intensity in CA3. *F*_(1,28)_ = 7.555, *p* < 0.05 for the effect on BDNF intensity in the hilus and arms of the dentate gyrus.

To determine whether the lack of an effect on the regrowth of 5-HT axons is specific to fluoxetine or if it can be generalized more broadly to SSRIs as a class, we tested a second SSRI. Following the same experimental design as our fluoxetine trial, we treated Slc6a4-GFP mice with 10 mg/kg sertraline once daily for 6 weeks after a challenge period with PCA or a saline control. A main effect of PCA on total axon length was present after 6 weeks of treatment (*F*_(1,62) _= 93.611, *p* < 0.0001; *F*_(1,31)_ = 84.883, *p* < 0.0001 for saline treated; *F*_(1,31)_ = 23.361, *p* < 0.0001 for sertraline treated; Fig. 5*A*,*B*). Similar to our fluoxetine trials, there was no significant main effect of treatment with 10 mg/kg sertraline (*F*_(1,62) _= 0.214; *p* = 0.645; Fig. 5*A*,*B*). Sertraline did not change total axon length in animals initially challenged with either PCA (*F*_(1,31)_ = 2.401; *p* = 0.131; Fig. 5*A*,*B*) or saline (*F*_(1,31)_ = 3.151; *p* = 0.086; Fig. 5*A*,*B*) compared with saline-treated controls.

Chronic treatment with sertraline has been shown to increase the expression of BDNF in the hippocampus of rodents (Nibuya et al., 1995; Peng et al., 2008; Fuchs et al., 2020). To validate our method of sertraline delivery, we measured the intensity of BDNF immunoreactivity in the hippocampus. Due to the diffuse staining pattern of BDNF, cell boundaries are difficult to define (Dieni et al., 2012; Braun et al., 2017; Andreska et al., 2020). For this reason, we measured the mean intensity of pixels in the anti-BDNF channel in maximally projected image stacks instead of counting individual cells like we did with DCX. Animals treated with sertraline had a significantly higher mean pixel intensity of BDNF staining in both the CA3 region (*F*_(1,28)_ = 11.302; *p* < 0.01; Fig. 5*C*,*D*) and in the hilus and blades of the dentate gyrus (*F*_(1,28)_ = 7.555; *p* < 0.05; Fig. 5*C*,*D*) compared with saline-treated controls. Taken together, the present results (Figs. 2–5) indicate that, following a chemical lesion of 5-HT axons with PCA, chronic treatment with two different SSRIs does not have an effect on 5-HT axon regrowth in the primary somatosensory cortex.

### Chronic fluoxetine or sertraline treatment does not affect 5-HT axon regrowth in adult mouse primary visual cortex

To verify that the lack of an effect of SSRIs on the regrowth of 5-HT axons can be generalized across the adult mouse cortex, we analyzed axon length in the primary visual cortex (V1). Using the same tissue analyzed in the previous figures, images of layer 1 of V1 were acquired, and total axon length was measured using exhaustive tracing of GFP-labeled fibers in 3D projected confocal stacks cropped to an equivalent 3D volume.

**Figure 6.**
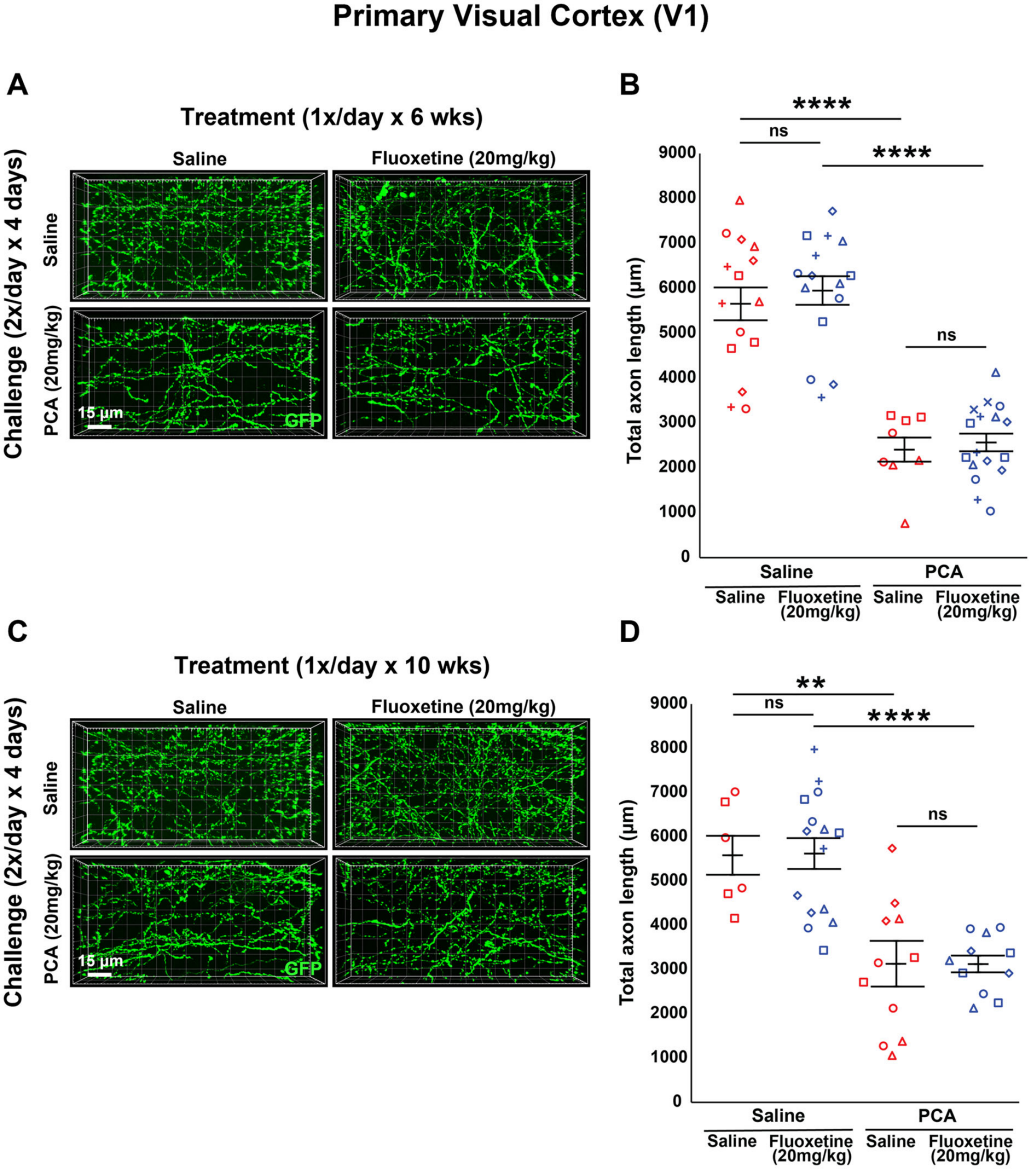
Daily injections of 20 mg/kg fluoxetine following injury with PCA do not affect 5-HT axon regrowth in layer 1 of primary visual cortex (V1). ***A***, ***B***, Slc6a4-GFP mice were initially treated with either PCA or saline for 4 d, allowed 2 d of recovery, and then followed by daily injections of either fluoxetine or saline for 6 weeks. ***A***, Representative images of each combination of conditions. ***B***, Total axon length was determined via histological analysis. Images were acquired using confocal microscopy and layer 1 of area V1 was analyzed in 3D with IMARIS software. Each animal had three technical replicates to account for within-animal variability. Each animal is represented by a different plot symbol. Mean and SE are plotted. Group data were analyzed using a mixed effect repeated-measures ANOVA. *N* = 9 males and 10 females. *F*_(1,29)_ = 74.3, *p* < 0.0001 for the effect of PCA in fluoxetine-treated animals. *F*_(1,22)_ = 36, *p* < 0.0001 for the effect of PCA in saline-treated animals. *F*_(1,23)_ = 0.202, *p* = 0.657 for the effect of fluoxetine in PCA-treated animals. *F*_(1,28)_ = 0.218, *p* = 0.644 for the effect of fluoxetine in saline-treated animals. ***C***, ***D***, Slc6a4-GFP mice were initially treated with either PCA or saline for 4 d, allowed 2 d of recovery, and then followed by daily injections of either fluoxetine or saline for 10 weeks. ***C***, Representative images of each combination of conditions. ***D***, Each animal is represented by a different plot symbol. Mean and SE are plotted. Group data were analyzed using a mixed effect repeated-measures ANOVA. *N* = 3 males and 12 females. *F*_(1,24)_ = 30.1, *p* < 0.0001 for the effect of PCA in fluoxetine-treated animals. *F*_(1,15)_ = 12.7, *p* < 0.01 for the effect of PCA in saline-treated animals. *F*_(1,20)_ = 0.028, *p* = 0.869 for the effect of fluoxetine in PCA-treated animals. *F*_(1,19)_ = 0.003, *p* = 0.954 for the effect of fluoxetine in saline-treated animals.

After 6 weeks of treatment with 20 mg/kg of fluoxetine, there was a main effect of PCA (*F*_(1,51) _= 102.741; *p* < 0.0001) where mice that received PCA during their challenge period had a significantly smaller total axon length compared with saline controls regardless of treatment (*F*_(1,22)_ = 36, *p* < 0.0001 for saline treated; *F*_(1,29)_ = 74.3, *p* < 0.0001 for fluoxetine treated; Fig. 6*A*,*B*). Similar to analysis in S1, there was not a main effect of 20 mg/kg of fluoxetine treatment for 6 weeks (*F*_(1,51)_ = 0.363; *p* = 0.549; Fig. 6*A*,*B*). Fluoxetine did not affect total axon length in animals initially challenged with PCA (*F*_(1,23)_ = 0.202; *p* = 0.657; Fig. 6*A*,*B*) or saline (*F*_(1,28)_ = 0.218; *p* = 0.644; Fig. 6*A*,*B*) compared with saline controls. Treatment with 20 mg/kg of fluoxetine for 10 weeks yielded similar results. A main effect of PCA was observed (*F*_(1,39) _= 39.019, *p* < 0.0001; *F*_(1,15)_ = 12.7, *p* < 0.01 for saline treated; *F*_(1,24)_ = 30.1, *p* < 0.0001 for fluoxetine treated; Fig. 6*C*,*D*), while there was not a main effect of 20 mg/kg fluoxetine (*F*_(1,39) _= 0.022, *p* = 0.882; *F*_(1,19)_ = 0.003, *p* = 0.954 for saline treated; *F*_(1,20)_ = 0.284, *p* = 0.869 for PCA treated; Fig. 6*C*,*D*).

**Figure 7.**
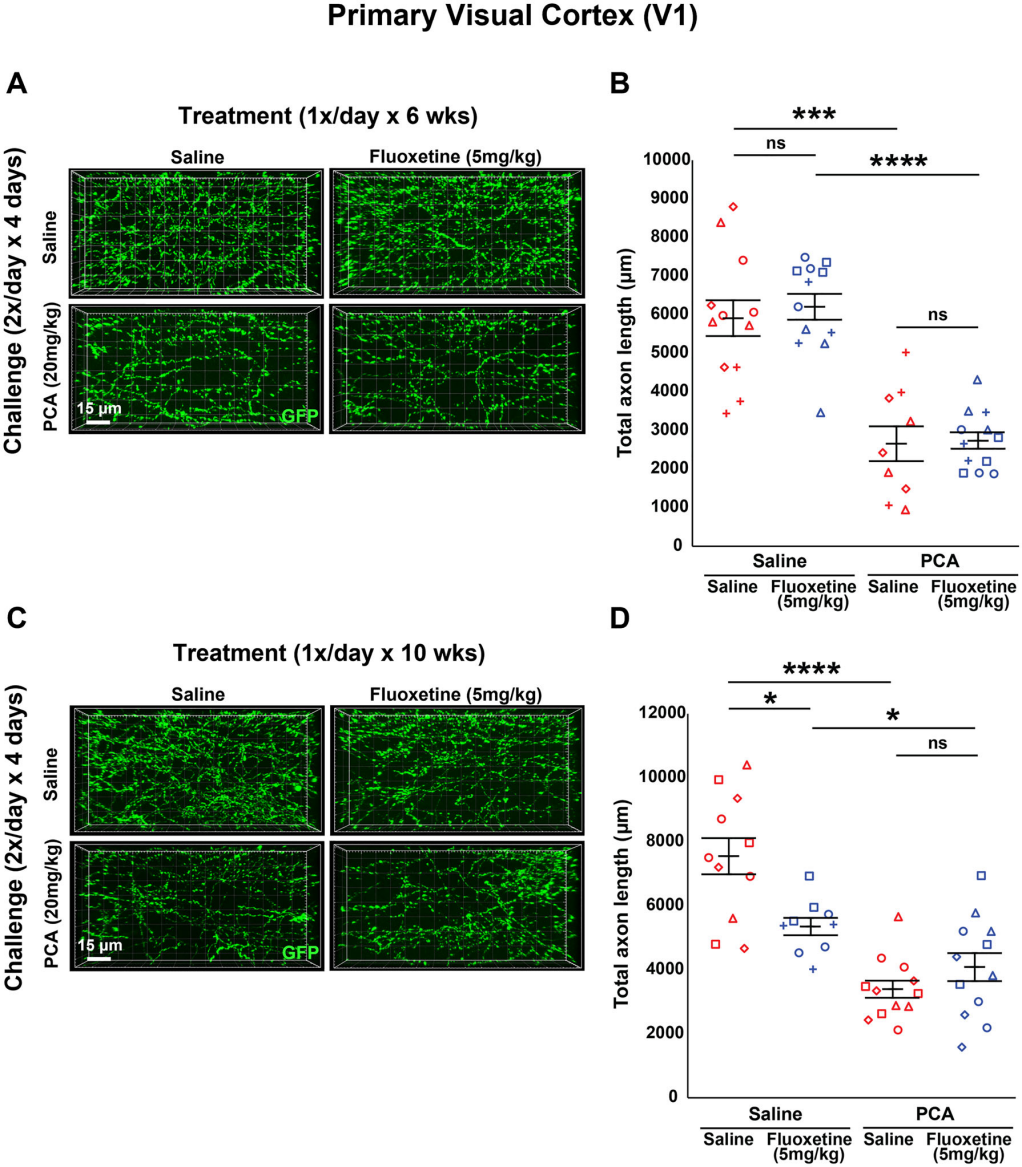
Daily injections of 5 mg/kg fluoxetine following injury with PCA do not affect 5-HT axon regrowth in layer 1 of primary visual cortex. ***A***, ***B***, Slc6a4-GFP mice were initially treated with either PCA or saline for 4 d, allowed 2 d of recovery, and then followed by daily injections of either fluoxetine or saline for 6 weeks. ***A***, Representative images of each combination of conditions. ***B***, Each animal is represented by a different plot symbol. Mean and SE are plotted. Group data were analyzed using a mixed effect repeated-measures ANOVA. *N* = 5 males and 10 females. *F*_(1,22)_ = 70.1, *p* < 0.0001 for the effect of PCA in fluoxetine-treated animals. *F*_(1,19)_ = 21.8, *p* < 0.001 for the effect of PCA in saline-treated animals. *F*_(1,19)_ = 0.029, *p* = 0.866 for the effect of fluoxetine in PCA-treated animals. *F*_(1,22)_ = 0.247, *p* = 0.624 for the effect of fluoxetine in saline-treated animals. ***C***, ***D***, Slc6a4-GFP mice were initially treated with either PCA or saline for 4 d, allowed 2 d of recovery, and then followed by daily injections of either fluoxetine or saline for 10 weeks. ***C***, Representative images of each combination of conditions. ***D***, Each animal is represented by a different plot symbol. Mean and SE are plotted. Group data were analyzed using a mixed effect repeated-measures ANOVA. *N* = 6 males and 9 females. *F*_(1,19)_ = 4.66, *p* < 0.05 for the effect of PCA in fluoxetine-treated animals. *F*_(1,21)_ = 42.4, *p* < 0.0001 for the effect of PCA in saline-treated animals. *F*_(1,22)_ = 1.66, *p* = 0.211 for the effect of fluoxetine in PCA-treated animals. *F*_(1,18)_ = 9.64, *p* = 0.006 for the effect of fluoxetine in saline-treated animals.

In trials using 5 mg/kg of fluoxetine, we still observed a main effect of PCA after 6 weeks of treatment (*F*_(1,41) _= 73.114, *p* < 0.0001; *F*_(1,19)_ = 21.8, *p* < 0.001 for saline treated; *F*_(1,22)_ = 70.1, *p* < 0.0001 for fluoxetine treated; Fig. 7*A*,*B*) and 10 weeks of treatment (*F*_(1,40) _= 38.535, *p* < 0.0001; *F*_(1,21)_ = 42.4, *p* < 0.0001 for saline treated; *F*_(1,19)_ = 4.66, *p* < 0.05 for fluoxetine treated; Fig. 7*C*,*D*). Once again, there was no main effect of treatment with 5 mg/kg fluoxetine after 6 weeks of treatment (*F*_(1,41) _= 0.233, *p* = 0.632; *F*_(1,22)_ = 0.247, *p* = 0.624 for saline treated; *F*_(1,19)_ = 0.029, *p* = 0.866 for PCA treated; Fig. 7*A*,*B*) or 10 weeks of treatment (*F*_(1,40) _= 2.973, *p* = 0.092; *F*_(1,18)_ = 9.64, *p* = 0.006 for saline treated; *F*_(1,22)_ = 1.66, *p* = 0.211 for PCA treated; Fig. 7*C*,*D*).

**Figure 8.**
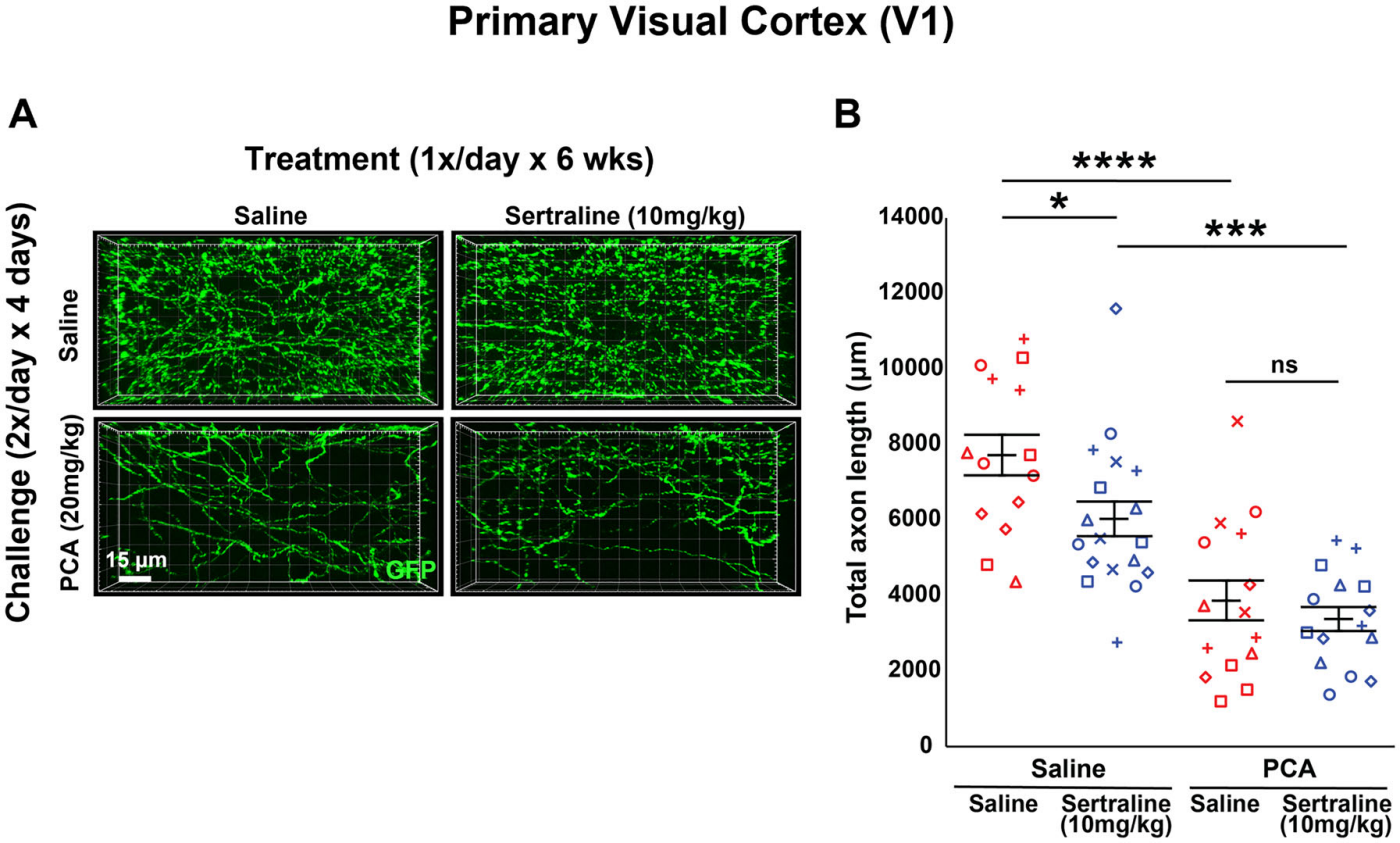
Daily injections of 10 mg/kg sertraline following injury with PCA do not affect 5-HT axon regrowth in layer 1 of primary visual cortex. ***A***, ***B***, Slc6a4-GFP mice were initially treated with either PCA or saline for 4 d, allowed 2 d of recovery, and then followed by daily injections of either sertraline or saline for 6 weeks. ***A***, Representative images of each combination of conditions. ***B***, Each animal is represented by a different plot symbol. Mean and SE are plotted. Group data were analyzed using a mixed effect repeated-measures ANOVA. *N* = 8 males and 14 females. *F*_(1,31)_ = 19.6, *p* < 0.001 for the effect of PCA in sertraline-treated animals. *F*_(1,27)_ = 24.4, *p* < 0.0001 for the effect of PCA in saline-treated animals. *F*_(1,28)_ = 0.592, *p* = 0.448 for the effect of sertraline in PCA-treated animals. *F*_(1,30)_ = 5.42, *p* = 0.027 for the effect of sertraline in saline-treated animals.

Analysis of total axon length in V1 after 6 weeks of treatment with 10 mg/kg of sertraline revealed comparable results. While there was a main effect of PCA (*F*_(1,58) _= 44.891, *p* < 0.0001; *F*_(1,27)_ = 24.4, *p* < 0.0001 for saline treated; *F*_(1,31)_ = 19.6, *p* < 0.001 for sertraline treated; Fig. 8*A*,*B*), a main effect of sertraline on PCA-treated animals was not observed (*F*_(1,58) _= 5.056, *p* = 0.028; *F*_(1,30)_ = 5.42, *p* = 0.027 for saline treated; *F*_(1,28)_ = 0.592, *p* = 0.448 for PCA treated; Fig. 8*A*,*B*). Collectively, the present results (Figs. 2–8) indicate that, following a chemical lesion of 5-HT axons with PCA, chronic treatment with two different SSRIs does not have a positive effect on 5-HT axon regrowth in the adult mouse cortex.

### Chronic fluoxetine or sertraline treatment does not affect 5-HT axon regrowth in adult mouse CA1 of the dorsal hippocampus

**Figure 9.**
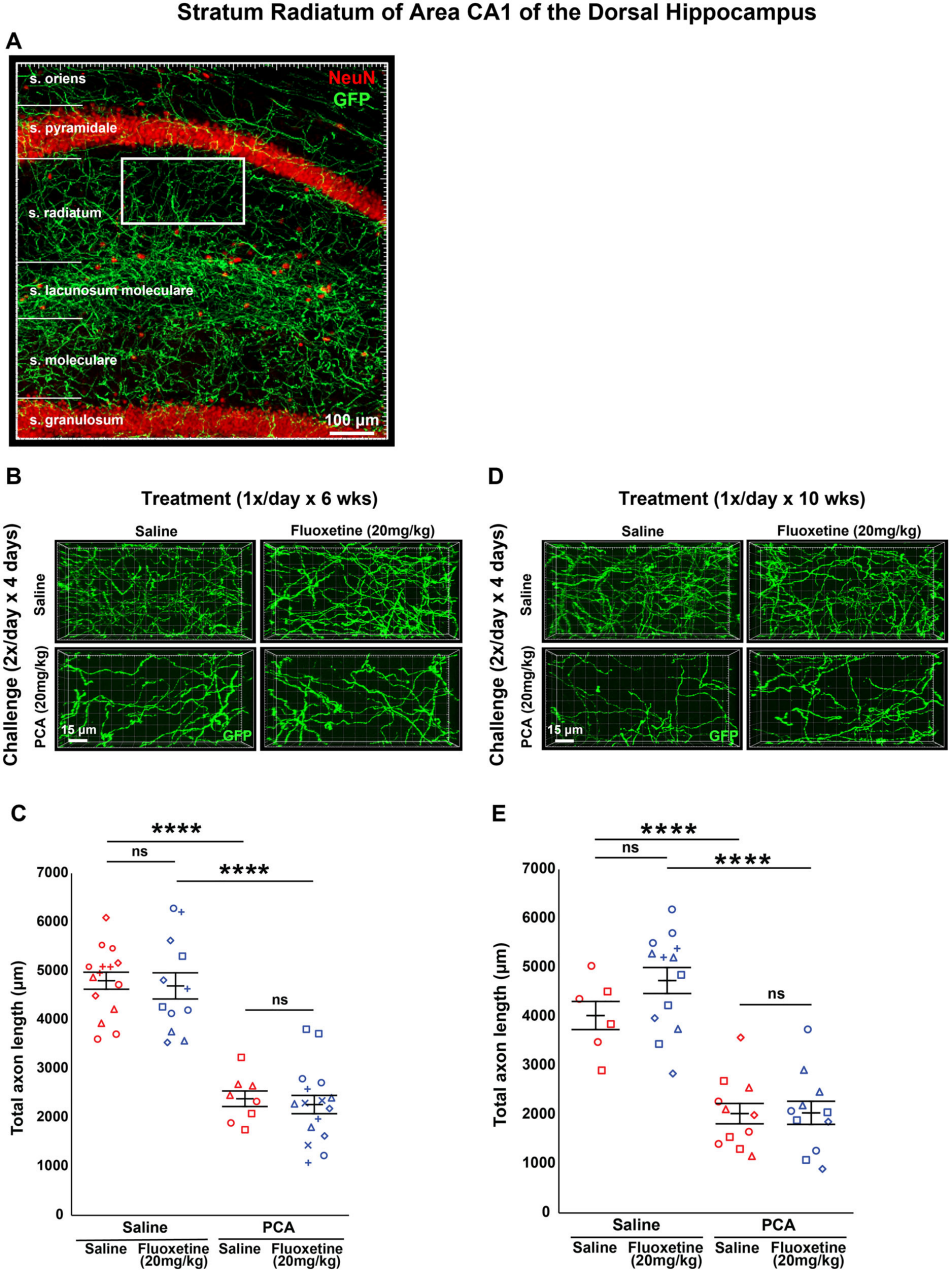
Daily injections of 20 mg/kg fluoxetine following injury with PCA do not affect 5-HT axon regrowth in area CA1 of the dorsal hippocampus. ***A***, Representative tiled confocal stack image of anti-GFP and anti-NeuN (neuronal nuclei marker) fluorescence of area CA1, with each layer indicated. An exemplary region of interest within the more dorsal portion of stratum radiatum is denoted by the white box. ***B***, ***C***, Slc6a4-GFP mice were initially treated with either PCA or saline for 4 d, allowed 2 d of recovery, and then followed by daily injections of either fluoxetine or saline for 6 weeks. ***B***, Representative images of each combination of conditions. ***C***, Total axon length was determined via histological analysis. Images were acquired using confocal microscopy and the stratum radiatum of the CA1 region of the hippocampus was analyzed in 3D with IMARIS software. Each animal had three technical replicates to account for within-animal variability. Each animal is represented by a different plot symbol. Mean and SE are plotted. Group data were analyzed using a mixed effect repeated-measures ANOVA. *N* = 9 males and 10 females. *F*_(1,26)_ = 54.5, *p* < 0.0001 for the effect of PCA in fluoxetine-treated animals. *F*_(1,21)_ = 75.4, *p* < 0.0001 for the effect of PCA in saline-treated animals. *F*_(1,22)_ = 0.152, *p* = 0.700 for the effect of fluoxetine in PCA-treated animals. *F*_(1,25)_ = 0.111, *p* = 0.742 for the effect of fluoxetine in saline-treated animals. ***D***, ***E***, Slc6a4-GFP mice were initially treated with either PCA or saline for 4 d, allowed 2 d of recovery, and then followed by daily injections of either fluoxetine or saline for 10 weeks. ***D***, Representative images of each combination of conditions. ***E***, Each animal is represented by a different plot symbol. Mean and SE are plotted. Group data were analyzed using a mixed effect repeated-measures ANOVA. *N* = 3 males and 12 females. *F*_(1,22)_ = 51.2, *p* < 0.0001 for the effect of PCA in fluoxetine-treated animals. *F*_(1,15)_ = 28.5, *p* < 0.0001 for the effect of PCA in saline-treated animals. *F*_(1,20)_ = 0.002, *p* = 0.962 for the effect of fluoxetine in PCA-treated animals. *F*_(1,17)_ = 2.39, *p* = 0.141 for the effect of fluoxetine in saline-treated animals.

All the analysis up to this point has been limited to the neocortex of the adult mouse. 5-HT axons broadly innervate the entire forebrain as illustrated in Figure 1*A*. To confirm that SSRIs do not influence 5-HT axon regrowth outside of cortical regions, we analyzed axon length in area CA1 of the dorsal hippocampus. More specifically, we chose a region of interest within the dorsal portion of the stratum radiatum of area CA1 (Fig. 9*A*). Once again, using the same tissue analyzed in the previous figures, images of stratum radiatum in area CA1 were acquired and total axon length was measured using exhaustive tracing of GFP-labeled fibers in 3D projected confocal stacks cropped to an equivalent 3D volume.

**Figure 10.**
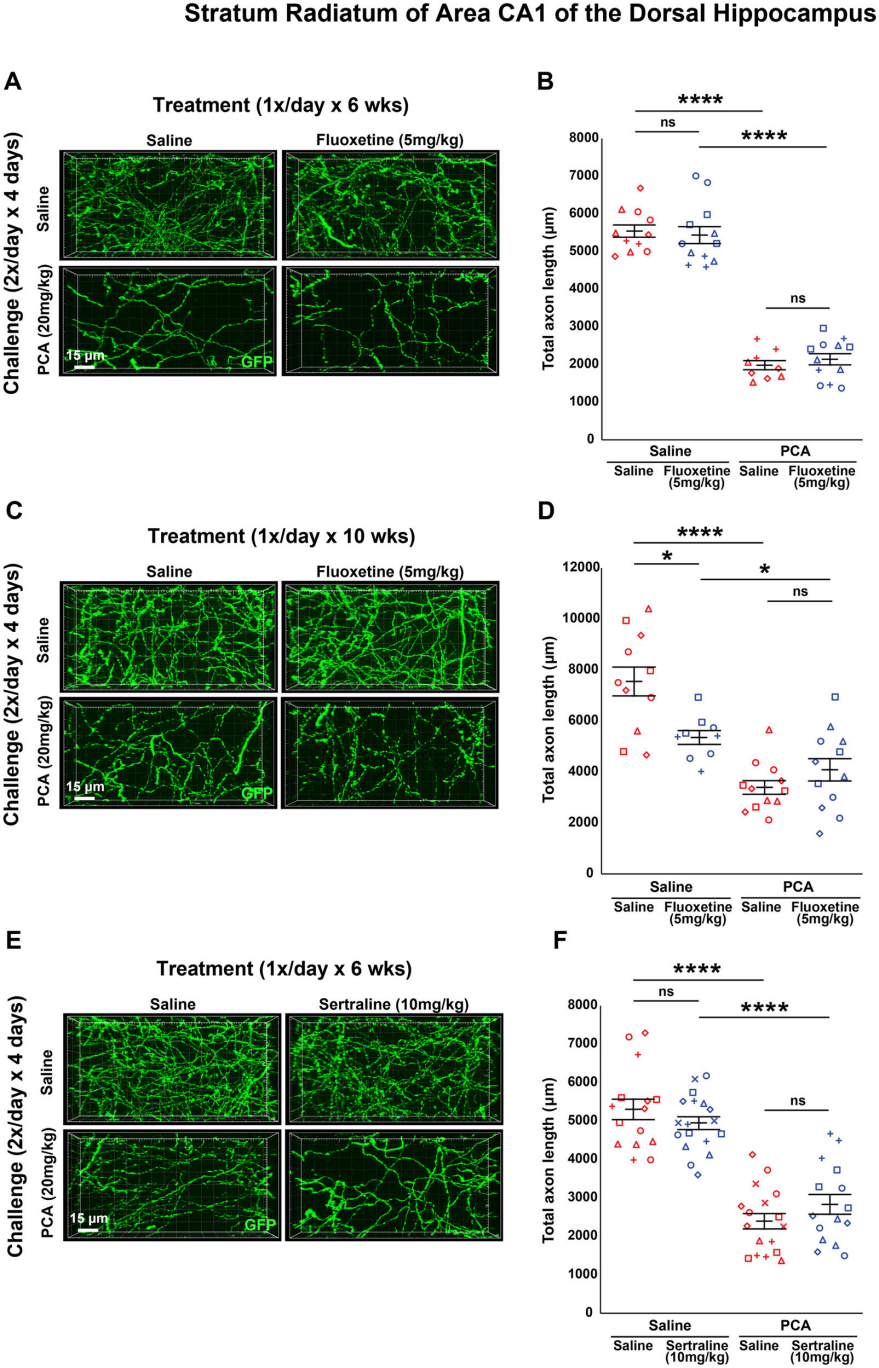
Daily injections of 5 mg/kg fluoxetine or 10 mg/kg sertraline following injury with PCA do not affect 5-HT axon regrowth in area CA1 of the dorsal hippocampus. ***A***, ***B***, Slc6a4-GFP mice were initially treated with either PCA or saline for 4 d, allowed 2 d of recovery, and then followed by daily injections of either fluoxetine or saline for 6 weeks. ***A***, Representative images of each combination of conditions. ***B***, Each animal is represented by a different plot symbol. Mean and SE are plotted. Group data were analyzed using a mixed effect repeated-measures ANOVA. *N* = 5 males and 10 females. *F*_(1,22)_ = 138, *p* < 0.0001 for the effect of PCA in fluoxetine-treated animals. *F*_(1,18)_ = 257, *p* < 0.0001 for the effect of PCA in saline-treated animals. *F*_(1,19)_ = 0.56, *p* = 0.463 for the effect of fluoxetine in PCA-treated animals. *F*_(1,21)_ = 0.13, *p* = 0.722 for the effect of fluoxetine in saline-treated animals. ***C***, ***E***, Slc6a4-GFP mice were initially treated with either PCA or saline for 4 d, allowed 2 d of recovery, and then followed by daily injections of either fluoxetine or saline for 10 weeks. ***C***, Representative images of each combination of conditions. ***D***, Each animal is represented by a different plot symbol. Mean and SE are plotted. Group data were analyzed using a mixed effect repeated-measures ANOVA. *N* = 6 males and 9 females. *F*_(1,19)_ = 41.8, *p* < 0.0001 for the effect of PCA in fluoxetine-treated animals. *F*_(1,22)_ = 65.9, *p* < 0.0001 for the effect of PCA in saline-treated animals. *F*_(1,22)_ = 0.088, *p* = 0.769 for the effect of fluoxetine in PCA-treated animals. *F*_(1,19)_ = 0.083, *p* = 0.776 for the effect of fluoxetine in saline-treated animals. ***E***, ***F***, Slc6a4-GFP mice were initially treated with either PCA or saline for 4 d, allowed 2 d of recovery, and then followed by daily injections of either sertraline or saline for 6 weeks. ***E***, Representative images of each combination of conditions. ***F***, Each animal is represented by a different plot symbol. Mean and SE are plotted. Group data were analyzed using a mixed effect repeated-measures ANOVA. *N* = 8 males and 14 females. *F*_(1,31)_ = 47.2, *p* < 0.0001 for the effect of PCA in sertraline-treated animals. *F*_(1,30)_ = 73.5, *p* < 0.0001 for the effect of PCA in saline-treated animals. *F*_(1,30)_ = 1.74, *p* = 0.196 for the effect of sertraline in PCA-treated animals. *F*_(1,31)_ = 1.28, *p* = 0.266 for the effect of sertraline in saline-treated animals.

We observed a main effect of PCA after treatment with 20 mg/kg of fluoxetine for both 6 weeks (*F*_(1,47) _= 117.649, *p* < 0.0001; *F*_(1,21)_ = 75.4, *p* < 0.0001 for saline treated; *F*_(1,26)_ = 54.5, *p* < 0.0001 for fluoxetine treated; Fig. 9*B*,*C*) and 10 weeks (*F*_(1,37) _= 71.606, *p* < 0.0001; *F*_(1,15)_ = 28.5, *p* < 0.0001 for saline treated; *F*_(1,22)_ = 51.2, *p* < 0.0001 for fluoxetine treated; Fig. 9*D*,*E*). However, there remained no main effect of 20 mg/kg fluoxetine on total axon length in stratum radiatum after either 6 weeks of treatment (*F*_(1,47) _= 0.252, *p* = 0.618; *F*_(1,25)_ = 0.111, *p* = 0.742 for saline treated; *F*_(1,22)_ = 0.152, *p* = 0.700 for fluoxetine treated; Fig. 9*B*,*C*) or 10 weeks of treatment (*F*_(1,37) _= 1.722, *p* = 0.197; *F*_(1,17)_ = 2.39, *p* = 0.141 for saline treated; *F*_(1,20)_ = 0.002, *p* = 0.962 for fluoxetine treated; Fig. 9*D*,*E*).

Trials with 5 mg/kg of fluoxetine and 10 mg/kg of sertraline yielded similar results to all our previously reported data (Figs. 2–9). There was a main effect of PCA after treatment with 5 mg/kg of fluoxetine for 6 weeks (*F*_(1,40) _= 345.043, *p* < 0.0001; *F*_(1,18)_ = 257, *p* < 0.0001 for saline treated; *F*_(1,22)_ = 138, *p* < 0.0001 for fluoxetine treated; Fig. 10*A*,*B*) and 10 weeks (*F*_(1,41) _= 105.082, *p* < 0.0001; *F*_(1,22)_ = 65.9, *p* < 0.0001 for saline treated; *F*_(1,19)_ = 41.8, *p* < 0.0001 for fluoxetine treated; Fig. 10*C*,*D*) and after treatment with 10 mg/kg of sertraline for 6 weeks (*F*_(1,61) _= 120.550, *p* < 0.0001; *F*_(1,30)_ = 73.5, *p* < 0.0001 for saline treated; *F*_(1,31)_ = 47.2, *p* < 0.0001 for sertraline treated; Fig. 10*E*,*F*). Nevertheless, there was not a main effect of 5 mg/kg fluoxetine after 6 weeks of treatment (*F*_(1,40) _= 0.0019, *p* = 0.891; *F*_(1,21)_ = 0.13, *p* = 0.722 for saline treated; *F*_(1,19)_ = 0.56, *p* = 0.463 for fluoxetine treated; Fig. 10*A*,*B*) or 10 weeks of treatment (*F*_(1,41) _= 0.000916, *p* = 0.976; *F*_(1,19)_ = 0.083, *p* = 0.722 for saline treated; *F*_(1,22)_ = 0.088, *p* = 0.769 for fluoxetine treated; Fig. 10*C*,*D*). There also was not a main effect of 10 mg/kg sertraline after 6 weeks of treatment (*F*_(1,61) _= 0.034, *p* = 0.855; *F*_(1,31)_ = 1.28, *p* = 0.266 for saline treated; *F*_(1,30)_ = 1.74, *p* = 0.196 for PCA treated; Fig. 10*E*,*F*). The combined outcomes (Figs. 2–10) suggest that chronic treatment with two different SSRIs does not have a positive effect on 5-HT axon regrowth in the adult mouse forebrain following a chemical lesion of 5-HT axons with PCA.

## Discussion

We investigated the effects of chronic (once daily) treatment with SSRIs on the regrowth of 5-HT axons in the mouse forebrain following a specific chemical lesion with PCA (Fig. 1), a treatment which has been shown to produce retrograde degeneration of 5-HT axons but which leaves 5-HT cell bodies intact (O’Hearn et al., 1988; Molliver et al., 1990; Wilson and Molliver, 1994; Jin et al., 2016). Once daily application of 20 mg/kg fluoxetine starting 3 d after the end of the PCA challenge period had no significant effect on 5-HT axon regrowth when applied for either 6 weeks (Figs. 2, 6, 9) or 10 weeks (Figs. 3, 6, 9) in adult Slc6a4-GFP mice. We also did not see a significant effect on 5-HT axon regrowth when we applied a lower dose of once daily 5 mg/kg fluoxetine for either 6 or 10 weeks (Figs. 4, 7, 10).

As a positive control, to ensure that our method of fluoxetine delivery was effective, we measured the number of DCX-positive cells in the hippocampal dentate gyrus (Navailles et al., 2008; Wang et al., 2011; Klomp et al., 2014; Sachs and Caron, 2014). Animals treated with fluoxetine at 5 or 10 mg/kg for either 6 or 10 weeks had significantly more DCX-positive cells normalized to the total length of the dentate gyrus compared with those treated with saline (Figs. 2–4). DCX immunoreactivity is often used as a marker for adult neurogenesis, but whether this interpretation is reliable in various mammalian species is an ongoing debate (Hagihara et al., 2019; La Rosa et al., 2020; Sorrells et al., 2021). For our purposes herein, we are not investigating adult neurogenesis and make no claims that this is or is not occurring; rather we have merely used increases in DCX immunoreactivity as an index for fluoxetine effects.

In addition, we tested a second SSRI in order to determine whether fluoxetine’s failure to alter 5-HT axon regrowth is a more general property of this class of drug. We found that treatment with a once daily 10 mg/kg sertraline dose for 6 weeks also resulted in no significant effect on 5-HT axon regrowth (Figs. 5, 8, 10). As a positive control for sertraline treatment, we used increases in anti-BDNF fluorescence intensity in the hippocampus as an indicator for sertraline effects (Nibuya et al., 1995; Peng et al., 2008; Fuchs et al., 2020; Fig. 5). In summary, we found no effects of chronic treatment with two different SSRIs on the regrowth of 5-HT axons in the adult mouse neocortex following PCA lesion.

These negative results do not support the use of SSRIs as a therapy following brain injury, but there are a few important caveats to consider. First, we only investigated the effect of chronic SSRI treatment on 5-HT axons. Most brain injuries, particularly physical injuries, are not selective in the class of neurons or axons which are damaged. SSRIs may affect the regrowth potential of other axonal types that mostly fail to regrow in the CNS (Cooke et al., 2022) like those that are glutamatergic or GABAergic. Alternatively, SSRIs might enhance the previously reported regrowth of noradrenergic axons, following chemical (Levin, 1983; Fritschy and Grzanna, 1992; Nakai et al., 1994) or physical (Pickel et al., 1973; Dougherty et al., 2020) injury. Second, we measured 5-HT axon regrowth only in layer 1 of S1, layer 1 of V1, and area CA1 of the dorsal hippocampus. There is a small possibility that PCA-evoked regrowth of 5-HT axons in other regions of the brain may be affected differently by chronic treatment with SSRIs. Finally, we only investigated the effects of SSRIs in one model of injury. Although treatment with PCA is selective to 5-HT axons, it results in extensive brain-wide retrograde degeneration of these axons to the first presynaptic active zone (Molliver et al., 1990; Fuller, 1992). Axons injured by a more localized injury such as one resulting from a stroke or a cortical impact (Kajstura et al., 2018) may respond differently to chronic treatment with SSRIs.

In a study most similar to ours, 11-week-old Pet-ChR-YFP mice (which express YFP exclusively in 5-HT neurons) were treated once daily with 18 mg/kg fluoxetine via drinking water for 6 weeks starting a week after an endothelin-1–induced stroke in the medial prefrontal cortex (Zahrai et al., 2020). A week after stroke, a loss of 5-HT fibers was observed in the cingulate gyrus and basolateral amygdala measured via costaining with YFP and SERT in fixed tissue. After 6 weeks, there was a reversal of the loss of 5-HT fibers in vehicle-treated mice, but this effect was more robust in animals that received fluoxetine. Researchers measured total axonal volume, rather than length, in a single slice per animal with three animals per condition. They also did not treat mice who received sham strokes with fluoxetine to determine any basal effects on 5-HT axon growth. These variations in experimental design, along with the type of injury induced and region of the brain examined, could account for our different outcomes.

Some clinical studies in humans have suggested a positive effect of treatment with SSRIs on functional recovery after stroke (Chollet et al., 2011; Mead et al., 2013; Gu and de Wang, 2018; Lin et al., 2018) and after TBI (Fann et al., 2001). However, two new meta-analyses (Legg et al., 2021; Hua et al., 2022), along with the completion of recent clinical trials (Yeo et al., 2019; Dennis et al., 2020; Hankey et al., 2020; Lundström et al., 2020; Almeida et al., 2021; Mateen et al., 2021), has indicated that chronic treatment with fluoxetine does not lead to any significant changes in poststroke recovery apart from decreased incidence of depression. However, acute administration of fluoxetine to rodents after stroke does facilitate recovery via reduction of infarct volumes (Windle and Corbett, 2005; Tae et al., 2009) and in some cases is accompanied by increased motor function and decreased neurological deficits (Lim et al., 2009). Certain studies even suggest that SSRIs initiated acutely after stroke establish an increased period of plasticity that leads to an increased response to motor training or structured physical rehabilitation (Ng et al., 2015; Alamri et al., 2021; Schneider et al., 2021).

Our study adds to the complex and divisive literature on functional improvements from chronic SSRI treatment after brain injury. Whether any positive effects seen from SSRI treatment result from a relief in depression and/or an increased period of plasticity leading to improved efforts in physical therapy remains to be determined. However, the present results indicate that any potential benefits of SSRI therapy after brain injury are unrelated to altering 5-HT axon regrowth.

## Synthesis

Reviewing Editor: Jeffery Twiss, University of South Carolina Decisions are customarily a result of the Reviewing Editor and the peer reviewers coming together and discussing their recommendations until a consensus is reached. When revisions are invited, a fact-based synthesis statement explaining their decision and outlining what is needed to prepare a revision will be listed below. The following reviewer(s) agreed to reveal their identity: Jerry Silver.

Both reviewers note that their concerns have been fully addressed.
